# Challenges of one-year longitudinal follow-up of a prospective, observational cohort study using an anonymised database: recommendations for trainee research collaboratives

**DOI:** 10.1186/s12874-019-0857-y

**Published:** 2019-12-12

**Authors:** STARSurg Collaborative, Sivesh Kamarajah, Kenneth A. McLean, Aditya Borakati, Thomas M. Drake, Evelina Woin, Chetan Khatri, J. Edward Fitzgerald, Ewen M. Harrison, Aneel Bhangu, Dmitri Nepogodiev, James C. Glasbey, Joshua Burke, Michael F. Bath, Henry A. Claireaux, Buket Gundogan, Midhun Mohan, Praveena Deekonda, Chia Kong, Holly Joyce, Lisa Mcnamee, Dmitri Nepogodiev, Nishkantha Arulkumaran, Samira Bell, Fiona Duthie, Jeremy Hughes, Thomas D. Pinkney, John Prowle, Toby Richards, Mark Thomas, K. Dynes, M. Patel, P. Patel, C. Wigley, R. Suresh, A. Shaw, S. Klimach, P. Jull, D. Evans, R. Preece, I. Ibrahim, V. Manikavasagar, R. Smith, F. S. Brown, P. Deekonda, R. Teo, D. P. Y. Sim, A. Borakati, A. E. Logan, I. Barai, H. Amin, S. Suresh, R. Sethi, W. Gul, W. Bolton, O. Corbridge, L. Horne, M. Attalla, R. Morley, C. Robinson, T. Hoskins, R. McAllister, S. Lee, Y. Dennis, G. Nixon, E. Heywood, H. Wilson, L. Ng, S. Samaraweera, A. Mills, C. Doherty, E. Woin, J. Belchos, V. Phan, M. Arnold, S. Sheik-Ali, R. Suresh, A. Cordaro, E. Mills, N. Lorch, D. Thomas, B. Ibrahim, S. Chee, T. Ngan, S. Pronin, N. Thakral, T. Yeoh, J. Wilson, R. Goodson, P. Molloy, M. Akhbari, W. Gul, R. Helliwell, S. Rees, M. Al-Attar, N. Griffiths, J. Mayes, P. Thomas, S. George, A. Thind, M. Kerr, F. Shafiq, I. Yasin, M. Gallagher, E. Sewart, T. Chouari, T. Gardner, N. Goergen, J. D. B. Hayes, C. S. MacLeod, R. McCormack, A. McKinley, S. McKinstry, W. Milligan, L. Ooi, N. M. Rafiq, T. Sammut, E. Sinclair, M. Smith, C. Baker, A. P. R. Boulton, J. Collins, H. C. Copley, N. Fearnhead, H. Fox, T. Mah, J. McKenna, V. Naruka, N. Nigam, B. Nourallah, S. Perera, A. Qureshi, S. Saggar, L. Sun, X. Wang, D. D. Yang, P. Caroll, C. Doyle, S. Elangovan, A. Falamarzi, K. Gascon Perai, E. Greenan, D. Jain, M. Lang-Orsini, S. Lim, L. O’Byrne, P. Ridgway, S. Van der Laan, J. Wong, J. Arthur, J. Barclay, P. Bradley, C. Edwin, E. Finch, E. Hayashi, M. Hopkins, D. Kelly, M. Kelly, N. McCartan, A. Ormrod, A. Pakenham, J. Hayward, C. Hitchen, A. Kishore, T. Martins, J. Philomen, R. Rao, C. Rickards, N. Burns, M. Copeland, C. Durand, A. Dyal, A. Ghaffar, A. Gidwani, M. Grant, C. Gribbon, A. Gruhn, M. Leer, K. Ahmad, G. Beattie, M. Beatty, G. Campbell, G. Donaldson, S. Graham, D. Holmes, S. Kanabar, H. Liu, C. McCann, R. Stewart, S. Vara, O. Ajibola-Taylor, E. J. E. Andah, C. Ani, N. M. O. Cabdi, G. Ito, M. Jones, A. Komoriyama, P. Patel, L. Titu, M. Basra, P. Gallogly, G. Harinath, S. H. Leong, A. Pradhan, I. Siddiqui, S. Zaat, A. Ali, M. Galea, W. L. Looi, J. C. K. Ng, G. Atkin, A. Azizi, Z. Cargill, Z. China, J. Elliot, R. Jebakumar, J. Lam, G. Mudalige, C. Onyerindu, M. Renju, V. Shankar Babu, M. Hussain, N. Joji, B. Lovett, H. Mownah, B. Ali, B. Cresswell, A. K. Dhillon, Y. S. Dupaguntla, C. Hungwe, J. D. Lowe-Zinola, J. C. H. Tsang, K. Bevan, C. Cardus, A. Duggal, S. Hossain, M. McHugh, M. Scott, F. Chan, R. Evans, E. Gurung, B. Haughey, B. Jacob-Ramsdale, M. Kerr, J. Lee, E. McCann, K. O’Boyle, N. Reid, F. Hayat, S. Hodgson, R. Johnston, W. Jones, M. Khan, T. Linn, S. Long, P. Seetharam, S. Shaman, B. Smart, A. Anilkumar, J. Davies, J. Griffith, B. Hughes, Y. Islam, D. Kidanu, N. Mushaini, I. Qamar, H. Robinson, M. Schramm, C. Yan Tan, H. Apperley, C. Billyard, J. M. Blazeby, S. P. Cannon, S. Carse, A. Göpfert, A. Loizidou, J. Parkin, E. Sanders, S. Sharma, G. Slade, R. Telfer, I. Whybrow Huppatz, E. Worley, L. Chandramoorthy, C. Friend, L. Harris, P. Jain, M. J. Karim, K. Killington, J. McGillicuddy, C. Rafferty, N. Rahunathan, T. Rayne, Y. Varathan, N. Verma, D. Zanichelli, M. Arneill, F. Brown, B. Campbell, L. Crozier, J. Henry, C. McCusker, P. Prabakaran, R. Wilson, U. Asif, M. Connor, S. Dindyal, N. Math, A. Pagarkar, H. Saleem, I. Seth, S. Sharma, N. Standfield, T. Swartbol, R. Adamson, J. E. Choi, O. El Tokhy, W. Ho, N. R. Javaid, M. Kelly, A. S. Mehdi, D. Menon, I. Plumptre, S. Sturrock, J. Turner, O. Warren, E. Crane, B. Ferris, C. Gadsby, J. Smallwood, M. Vipond, V. Wilson, T. Amarnath, A. Doshi, C. Gregory, K. Kandiah, B. Powell, H. Spoor, C. Toh, R. Vizor, M. Common, K. Dunleavy, S. Harris, C. Luo, Z. Mesbah, A. Prem Kumar, A. Redmond, S. Skulsky, T. Walsh, D. Daly, L. Deery, E. Epanomeritakis, M. Harty, D. Kane, K. Khan, R. Mackey, J. McConville, K. McGinnity, G. Nixon, A. Ang, J. Y. Kee, E. Leung, S. Norman, S. V. Palaniappan, P. Partha Sarathy, T. Yeoh, J. Frost, P. Hazeldine, L. Jones, M. Karbowiak, C. Macdonald, A. Mutarambirwa, A. Omotade, M. Runkel, G. Ryan, N. Sawers, C. Searle, S. Suresh, S. Vig, A. Ahmad, R. McGartland, R. Sim, A. Song, J. Wayman, R. Brown, L. H. Chang, K. Concannon, C. Crilly, T. J. Arnold, A. Burgin, F. Cadden, C. H. Choy, M. Coleman, D. Lim, J. Luk, P. Mahankali-Rao, A. J. Prudence-Taylor, D. Ramakrishnan, J. Russell, A. Fawole, J. Gohil, B. Green, A. Hussain, L. McMenamin, L. McMenamin, M. Tang, F. Azmi, S. Benchetrit, T. Cope, A. Haque, A. Harlinska, R. Holdsworth, T. Ivo, J. Martin, T. Nisar, A. Patel, K. Sasapu, J. Trevett, G. Vernet, A. Aamir, C. Bird, A. Durham-Hall, W. Gibson, J. Hartley, N. May, V. Maynard, S. Johnson, C. Mc Donald Wood, M. O’Brien, J. Orbell, T. D. Stringfellow, F. Tenters, S. Tresidder, W. Cheung, A. Grant, N. Tod, M. Bews-Hair, Z. H. Lim, S. W. Lim, M. Vella-Baldacchino, S. Auckburally, A. Chopada, S. Easdon, R. Goodson, F. McCurdie, M. Narouz, A. Radford, E. Rea, O. Taylor, T. Yu, M. Alfa-Wali, L. Amani, I. Auluck, P. Bruce, J. Emberton, R. Kumar, N. Lagzouli, A. Mehta, A. Murtaza, M. Raja, I. S. Dennahy, K. Frew, A. Given, Y. Y. He, M. A. Karim, E. MacDonald, E. McDonald, D. McVinnie, S. K. Ng, A. Pettit, D. P. Y. Sim, S. D. Berthaume-Hawkins, R. Charnley, K. Fenton, D. Jones, C. Murphy, J. Q. Ng, R. Reehal, H. Robinson, S. S. Seraj, E. Shang, A. Tonks, P. White, A. Yeo, P. Chong, R. Gabriel, N. Patel, E. Richardson, L. Symons, D. Aubrey-Jones, S. Dawood, M. Dobrzynska, S. Faulkner, H. Griffiths, F. Mahmood, P. Patel, M. Perry, A. Power, R. Simpson, A. Ali, P. Brobbey, A. Burrows, P. Elder, R. Ganyani, C. Horseman, P. Hurst, H. Mann, K. Marimuthu, S. McBride, E. Pilsworth, N. Powers, P. Stanier, R. Innes, T. Kersey, M. Kopczynska, N. Langasco, N. Patel, R. Rajagopal, B. Atkins, W. Beasley, Z. Cheng Lim, A. Gill, H. Li Ang, H. Williams, T. Yogeswara, R. Carter, M. Fam, J. Fong, J. Latter, M. Long, S. Mackinnon, C. McKenzie, J. Osmanska, V. Raghuvir, A. Shafi, K. Tsang, L. Walker, K. Bountra, O. Coldicutt, D. Fletcher, S. Hudson, S. Iqbal, T. Lopez Bernal, J. W. B. Martin, F. Moss-Lawton, J. Smallwood, A. Cardwell, K. Edgerton, J. Laws, A. Rai, K. Robinson, K. Waite, J. Ward, H. Youssef, C. Knight, P. Y. Koo, A. Lazarou, S. Stanger, C. Thorn, M. C. Triniman, A. Botha, L. Boyles, S. Cumming, S. Deepak, A. Ezzat, A. J. Fowler, A. M. Gwozdz, S. F. Hussain, S. Khan, H. Li, B. Lu Morrell, J. Neville, R. Nitiahpapand, O. Pickering, H. Sagoo, E. Sharma, K. Welsh, S. Denley, S. Khan, M. Agarwal, N. Al-Saadi, R. Bhambra, A. Gupta, Z. A. R. Jawad, L. R. Jiao, K. Khan, G. Mahir, S. Singagireson, B. L. Thoms, B. Tseu, R. Wei, N. Yang, N. Britton, D. Leinhardt, M. Mahfooz, A. Palkhi, M. Price, S. Sheikh, M. Barker, D. Bowley, M. Cant, U. Datta, M. Farooqi, A. Lee, G. Morley, M. Naushad Amin, A. Parry, S. Patel, S. Strang, N. Yoganayagam, A. Adlan, S. Chandramoorthy, Y. Choudhary, K. Das, M. Feldman, B. France, R. Grace, H. Puddy, P. Soor, M. Ali, P. Dhillon, A. Faraj, L. Gerard, M. Glover, H. Imran, S. Kim, Y. Patrick, J. Peto, A. Prabhudesai, R. Smith, A. Tang, N. Vadgama, R. Dhaliwal, T. Ecclestone, A. Harris, D. Ong, D. Patel, C. Philp, E. Stewart, L. Wang, E. Wong, Y. Xu, T. Ashaye, T. Fozard, F. Galloway, S. Kaptanis, P. Mistry, T. Nguyen, F. Olagbaiye, M. Osman, Z. Philip, R. Rembacken, S. Tayeh, K. Theodoropoulou, A. Herman, J. Lau, A. Saha, M. Trotter, O. Adeleye, D. Cave, T. Gunwa, J. Magalhães, S. Makwana, R. Mason, M. Parish, H. Regan, P. Renwick, G. Roberts, D. Salekin, C. Sivakumar, A. Tariq, I. Liew, A. McDade, D. Stewart, M. Hague, N. Hudson-Peacock, C. E. S. Jackson, F. James, J. Pitt, E. Y. Walker, R. Aftab, J. J. Ang, S. Anwar, J. Battle, E. Budd, J. Chui, H. Crook, P. Davies, S. Easby, E. Hackney, B. Ho, S. Z. Imam, J. Rammell, H. Andrews, C. Perry, P. Schinle, P. Ahmed, T. Aquilina, E. Balai, M. Church, E. Cumber, A. Curtis, G. Davies, Y. Dennis, E. Dumann, S. Greenhalgh, P. Kim, S. King, K. H. M. Metcalfe, L. Passby, N. Redgrave, Z. Soonawalla, S. Waters, A. Zornoza, I. Gulzar, J. Hole, K. Hull, H. Ishaq, J. Karaj, A. Kelkar, E. Love, S. Patel, D. Thakrar, M. Vine, A. Waterman, N. P. Dib, N. Francis, M. Hanson, R. Ingleton, K. S. Sadanand, N. Sukirthan, S. Arnell, M. Ball, N. Bassam, G. Beghal, A. Chang, V. Dawe, A. George, T. Huq, A. Hussain, B. Ikram, L. Kanapeckaite, M. Khan, D. Ramjas, A. Rushd, S. Sait, M. Serry, E. Yardimci, S. Capella, L. Chenciner, C. Episkopos, E. Karam, C. McCarthy, W. Moore-Kelly, N. Watson, V. Ahluwalia, J. Barnfield, O. Ben-Gal, I. Bloom, A. Gharatya, K. Khodatars, N. Merchant, A. Moonan, M. Moore, K. Patel, H. Spiers, K. Sundaram, J. Turner, M. F. Bath, J. Black, H. Chadwick, L. Huisman, H. Ingram, S. Khan, L. Martin, M. Metcalfe, P. Sangal, J. Seehra, A. Thatcher, S. Venturini, I. Whitcroft, Z. Afzal, S. Brown, A. Gani, A. Gomaa, N. Hussein, S. Y. Oh, N. Pazhaniappan, E. Sharkey, T. Sivagnanasithiyar, C. Williams, J. Yeung, L. Cruddas, S. Gurjar, A. Pau, R. Prakash, R. Randhawa, L. Chen, I. Eiben, M. Naylor, D. Osei-Bordom, R. Trenear, J. Bannard-Smith, N. Griffiths, B. Y. Patel, F. Saeed, H. Abdikadir, M. Bennett, R. Church, S. E. Clements, J. Court, A. Delvi, J. Hubert, B. Macdonald, F. Mansour, R. R. Patel, R. Perris, S. Small, A. Betts, N. Brown, A. Chong, C. Croitoru, A. Grey, P. Hickland, C. Ho, D. Hollington, L. McKie, A. R. Nelson, H. Stewart, P. Eiben, M. Nedham, I. Ali, T. Brown, S. Cumming, C. Hunt, C. Joyner, C. McAlinden, J. Roberts, D. Rogers, A. Thachettu, N. Tyson, R. Vaughan, N. Verma, T. Yasin, K. Andrew, N. Bhamra, S. Leong, R. Mistry, H. Noble, F. Rashed, N. R. Walker, L. Watson, M. Worsfold, E. Yarham, H. Abdikadir, A. Arshad, B. Barmayehvar, L. Cato, N. Chan-lam, V. Do, A. Leong, Z. Sheikh, T. Zheleniakova, J. Coppel, S. T. Hussain, R. Mahmood, R. Nourzaie, J. Prowle, S. Sheik-Ali, A. Thomas, A. Alagappan, R. Ashour, H. Bains, J. Diamond, J. Gordon, B. Ibrahim, M. Khalil, D. Mittapalli, Y. N. Neo, P. Patil, F. S. Peck, N. Reza, I. Swan, M. Whyte, S. Chaudhry, J. Hernon, H. Khawar, J. O’Brien, M. Pullinger, K. Rothnie, S. Ujjal, S. Bhatte, J. Curtis, S. Green, A. Mayer, G. Watkinson, K. Chapple, T. Hawthorne, M. Khaliq, L. Majkowski, T. A. M. Malik, K. Mclauchlan, B. Ng Wei En, T. O’Coenenor, S. Parton, S. D. Robinson, M. I. Saat, B. N. Shurovi, K. Varatharasasingam, A. E. Ward, K. Behranwala, M. Bertelli, J. Cohen, F. Duff, O. Fafemi, R. Gupta, M. Manimaran, J. Mayhew, D. Peprah, M. H. Y. Wong, N. Farmer, C. Houghton, N. Kandhari, K. Khan, D. Ladha, J. Mayes, F. McLennan, P. Panahi, H. Seehra, R. Agrawal, I. Ahmed, S. Ali, F. Birkinshaw, M. Choudhry, S. Gokani, S. Harrogate, S. Jamal, F. Nawrozzadeh, A. Swaray, A. Szczap, J. Warusavitarne, M. Abdalla, N. Asemota, R. Cullum, M. Hartley, C. Maxwell-Armstrong, C. Mulvenna, J. Phillips, A. Yule, L. Ahmed, K. D. Clement, N. Craig, E. Elseedawy, D. Gorman, L. Kane, J. Livie, V. Livie, E. Moss, A. Naasan, F. Ravi, P. Shields, Y. Zhu, M. Archer, H. Cobley, R. Dennis, C. Downes, B. Guevel, E. Lamptey, H. Murray, A. Radhakrishnan, S. Saravanabavan, M. Sardar, C. Shaw, V. Tilliridou, R. Wright, W. Ye, N. Alturki, R. Helliwell, E. Jones, D. Kelly, S. Lambotharan, K. Scott, R. Sivakumar, L. Victor, H. Boraluwe-Rallage, P. Froggatt, S. Haynes, Y. M. A. Hung, A. Keyte, L. Matthews, E. Evans, P. Haray, I. John, A. Mathivanan, L. Morgan, O. Oji, C. Okorocha, A. Rutherford, H. Spiers, N. Stageman, A. Tsui, R. Whitham, A. Amoah-Arko, E. Cecil, A. Dietrich, H. Fitzpatrick, C. Guy, J. Hair, J. Hilton, L. Jawad, E. McAleer, Z. Taylor, J. Yap, M. Akhbari, D. Debnath, T. Dhir, M. Elbuzidi, M. Elsaddig, S. Glace, H. Khawaja, R. Koshy, K. Lal, L. Lobo, A. McDermott, J. Meredith, M. A. Qamar, A. Vaidya, F. Acquaah, L. Barfi, N. Carter, D. Gnanappiragasam, C. Ji, F. Kaminski, S. Lawday, K. Mackay, S. K. Sulaiman, R. Webb, P. Ananthavarathan, F. Dalal, E. Farrar, R. Hashemi, M. Hossain, J. Jiang, M. Kiandee, J. Lex, L. Mason, J. H. Matthews, E. McGeorge, S. Modhwadia, T. Pinkney, A. Radotra, L. Rickard, L. Rodman, A. Sales, K. L. Tan, A. Bachi, D. S. Bajwa, J. Battle, L. R. Brown, A. Butler, A. Calciu, E. Davies, I. Gardner, T. Girdlestone, O. Ikogho, G. Keelan, P. O’Loughlin, J. Tam, J. Elias, M. Ngaage, J. Thompson, S. Bristow, E. Brock, H. Davis, M. Pantelidou, A. Sathiyakeerthy, K. Singh, A. Chaudhry, G. Dickson, P. Glen, K. Gregoriou, H. Hamid, A. Mclean, P. Mehtaji, G. Neophytou, S. Potts, D. R. Belgaid, J. Burke, J. Durno, N. Ghailan, M. Hanson, V. Henshaw, U. R. Nazir, I. Omar, B. J. Riley, J. Roberts, G. Smart, K. Van Winsen, A. Bhatti, M. Chan, M. D’Auria, S. Green, C. Keshvala, H. Li, C. Maxwell-Armstrong, M. Michaelidou, L. Simmonds, C. Smith, A. Wimalathasan, J. Abbas, C. Cairns, Y. R. Chin, A. Connelly, S. Moug, A. Nair, D. Svolkinas, P. Coe, D. Subar, H. Wang, V. Zaver, J. Brayley, P. Cookson, L. Cunningham, A. Gaukroger, M. Ho, A. Hough, J. King, D. O’Hagan, A. Widdison, R. Brown, B. Brown, A. Chavan, S. Francis, L. Hare, J. Lund, N. Malone, B. Mavi, A. McIlwaine, S. Rangarajan, N. Abuhussein, H. S. Campbell, J. Daniels, I. Fitzgerald, S. Mansfield, A. Pendrill, D. Robertson, Y. W. Smart, T. Teng, J. Yates, A. Belgaumkar, A. Katira, J. Kossoff, S. Kukran, C. Laing, B. Mathew, T. Mohamed, S. Myers, R. Novell, B. L. Phillips, M. Thomas, T. Turlejski, S. Turner, M. Varcada, L. Warren, W. Wynell-Mayow, R. Church, L. Linley-Adams, G. Osborn, M. Saunders, R. Spencer, M. Srikanthan, S. Tailor, A. Tullett, M. Ali, S. Al-Masri, G. Carr, O. Ebhogiaye, S. Heng, S. Manivannan, J. Manley, L. E. McMillan, C. Peat, B. Phillips, S. Thomas, H. Whewell, G. Williams, A. Bienias, E. A. Cope, G. R. Courquin, L. Day, C. Garner, A. Gimson, C. Harris, K. Markham, T. Moore, T. Nadin, C. Phillips, S. M. Subratty, K. Brown, J. Dada, M. Durbacz, T. Filipescu, E. Harrison, E. D. Kennedy, E. Khoo, D. Kremel, I. Lyell, S. Pronin, R. Tummon, C. Ventre, L. Walls, E. Wootton, A. Akhtar, E. Davies, D. El-Sawy, M. Farooq, M. Gaddah, H. Griffiths, I. Katsaiti, N. Khadem, K. Leong, I. Williams, C. S. Chean, D. Chudek, H. Desai, N. Ellerby, A. Hammad, S. Malla, B. Murphy, O. Oshin, P. Popova, S. Rana, T. Ward, T. E. F. Abbott, O. Akpenyi, F. Edozie, R. El Matary, W. English, S. Jeyabaladevan, C. Morgan, V. Naidu, K. Nicholls, S. Peroos, J. Prowle, S. Sansome, H. D. Torrance, D. Townsend, J. Brecher, H. Fung, Z. Kazmi, P. Outlaw, K. Pursnani, N. Ramanujam, A. Razaq, M. Sattar, S. Sukumar, T. S. E. Tan, K. Chohan, S. Dhuna, T. Haq, S. Kirby, J. Lacy-Colson, P. Logan, Q. Malik, J. McCann, Z. Mughal, S. Sadiq, I. Sharif, C. Shingles, A. Simon, S. Burnage, S. S. N. Chan, A. R. J. Craig, J. Duffield, A. Dutta, M. Eastwood, F. Iqbal, F. Mahmood, W. Mahmood, C. Patel, A. Qadeer, A. Robinson, A. Rotundo, A. Schade, R. D. Slade, M. De Freitas, H. Kinnersley, E. McDowell, S. Moens-Lecumberri, J. Ramsden, T. Rockall, L. Wiffen, S. Wright, C. Bruce, V. Francois, K. Hamdan, C. Limb, A. J. Lunt, L. Manley, M. Marks, C. F. E. Phillips, C. J. F. Agnew, C. J. Barr, N. Benons, S. J. Hart, D. Kandage, R. Krysztopik, P. Mahalingam, J. Mock, S. Rajendran, M. T. Stoddart, B. Clements, H. Gillespie, S. Lee, R. McDougall, C. Murray, R. O’Loane, S. Periketi, S. Tan, R. Amoah, R. Bhudia, B. Dudley, A. Gilbert, B. Griffiths, H. Khan, N. McKigney, B. Roberts, R. Samuel, A. Seelarbokus, A. Stubbing-Moore, G. Thompson, P. Williams, N. Ahmed, R. Akhtar, E. Chandler, I. Chappelow, H. Gil, T. Gower, A. Kale, G. Lingam, L. Rutler, C. Sellahewa, A. Sheikh, H. Stringer, R. Taylor, H. Aglan, M. R. Ashraf, S. Choo, E. Das, J. Epstein, R. Gentry, D. Mills, Y. Poolovadoo, N. Ward, K. Bull, A. Cole, J. Hack, S. Khawari, C. Lake, T. Mandishona, R. Perry, S. Sleight, S. Sultan, T. Thornton, S. Williams, T. Arif, A. Castle, P. Chauhan, R. Chesner, T. Eilon, S. Kamarajah, C. Kambasha, L. Lock, T. Loka, F. Mohammad, S. Motahariasl, L. Roper, S. S. Sadhra, A. Sheikh, T. Toma, Q. Wadood, J. Yip, E. Ainger, S. Busti, L. Cunliffe, T. Flamini, S. Gaffing, C. Moorcroft, M. Peter, L. Simpson, E. Stokes, G. Stott, J. Wilson, J. York, A. Yousaf, A. Borakati, M. Brown, A. Goaman, B. Hodgson, A. Ijeomah, U. Iroegbu, G. Kaur, C. Lowe, S. Mahmood, Z. Sattar, P. Sen, A. Szuman, N. Abbas, M. Al-Ausi, N. Anto, R. Bhome, L. Eccles, J. Elliott, E. J. Hughes, A. Jones, A. S. Karunatilleke, J. S. Knight, C. C. F. Manson, I. Mekhail, L. Michaels, T. M. Noton, E. Okenyi, T. Reeves, I. H. Yasin, D. A. Banfield, R. Harris, D. Lim, C. Mason-Apps, T. Roe, J. Sandhu, N. Shafiq, E. Stickler, J. P. Tam, L. M. Williams, P. Ainsworth, Y. Boualbanat, C. Doull, E. Egan, L. Evans, K. Hassanin, G. Ninkovic-Hall, W. Odunlami, M. Shergill, M. Traish, D. Cummings, S. Kershaw, J. Ong, F. Reid, H. Toellner, A. Alwandi, M. Amer, D. George, K. Haynes, K. Hughes, L. Peakall, Y. Premakumar, N. Punjabi, A. Ramwell, H. Sawkins, J. Ashwood, A. Baker, C. Baron, I. Bhide, E. Blake, C. De Cates, R. Esmail, H. Hosamuddin, J. Kapp, N. Nguru, M. Raja, F. Thomson, H. Ahmed, G. Aishwarya, R. Al-Huneidi, S. Ali, R. Aziz, D. Burke, B. Clarke, A. Kausar, D. Maskill, L. Mecia, L. Myers, A. C. D. Smith, G. Walker, N. Wroe, C. Donohoe, D. Gibbons, P. Jordan, C. Keogh, A. Kiely, P. Lalor, M. McCrohan, C. Powell, M. Power Foley, J. Reynolds, E. Silke, O. Thorpe, J. Tseun Han Kong, C. White, Q. Ali, J. Dalrymple, Y. Ge, H. Khan, R. S. Luo, H. Paine, B. Paraskeva, L. Parker, K. Pillai, J. Salciccioli, S. Selvadurai, V. Sonagara, L. R. Springford, L. Tan, S. Appleton, N. Leadholm, Y. Zhang, D. Ahern, M. Cotter, S. Cremen, T. Durrigan, V. Flack, N. Hrvacic, H. Jones, B. Jong, K. Keane, P. R. O’Connell, J. O’sullivan, G. Pek, S. Shirazi, C. Barker, A. Brown, W. Carr, Y. Chen, C. Guillotte, J. Harte, A. Kokayi, K. Lau, S. McFarlane, S. Morrison, J. Broad, N. Kenefick, D. Makanji, V. Printz, R. Saito, O. Thomas, H. Breen, S. Kirk, C. H. Kong, A. O’Kane, M. Eddama, A. Engledow, S. K. Freeman, A. Frost, C. Goh, G. Lee, R. Poonawala, A. Suri, P. Taribagil, H. Brown, S. Christie, S. Dean, R. Gravell, E. Haywood, F. Holt, E. Pilsworth, R. Rabiu, H. W. Roscoe, S. Shergill, A. Sriram, A. Sureshkumar, L. C. Tan, A. Tanna, A. Vakharia, S. Bhullar, S. Brannick, E. Dunne, M. Frere, M. Kerin, K. Muthu Kumar, T. Pratumsuwan, R. Quek, M. Salman, N. Van Den Berg, C. Wong, J. Ahluwalia, R. Bagga, C. M. Borg, C. Calabria, A. Draper, M. Farwana, H. Joyce, A. Khan, M. Mazza, G. Pankin, M. S. Sait, N. Sandhu, N. Virani, J. Wong, K. Woodhams, N. Croghan, S. Ghag, G. Hogg, O. Ismail, N. John, K. Nadeem, M. Naqi, S. M. Noe, A. Sharma, S. Tan, F. Begum, R. Best, A. Collishaw, J. Glasbey, D. Golding, B. Gwilym, P. Harrison, T. Jackman, N. Lewis, Y. L. Luk, T. Porter, S. Potluri, M. Stechman, S. Tate, B. Walford, F. Auld, A. Bleakley, S. Johnston, C. Jones, J. Khaw, S. Milne, S. O’Neill, K. K. R. Singh, R. Smith, A. Swan, N. Thorley, S. Yalamarthi, Z. D. Yin, A. Ali, V. Balian, R. Bana, K. Clark, C. Livesey, G. McLachlan, M. Mohammad, N. Pranesh, C. Richards, F. Ross, M. Sajid, M. Brooke, J. Francombe, J. Gresly, S. Hutchinson, K. Kerrigan, E. Matthews, S. Nur, L. Parsons, A. Sandhu, M. Vyas, F. White, A. Zulkifli, L. Zuzarte, A. Al-Mousawi, J. Arya, S. Azam, A. Azri Yahaya, K. Gill, R. Hallan, C. Hathaway, I. Leptidis, L. McDonagh, S. Mitrasinovic, N. Mushtaq, N. Pang, G. B. Peiris, S. Rinkoff, L. Chan, E. Christopher, M. M. H. Farhan-Alanie, A. Gonzalez-Ciscar, C. J. Graham, H. Lim, K. A. McLean, H. M. Paterson, A. Rogers, C. Roy, D. Rutherford, F. Smith, G. Zubikarai, R. Al-Khudairi, M. Bamford, M. Chang, J. Cheng, C. Hedley, R. Joseph, B. Mitchell, S. Perera, L. Rothwell, A. Siddiqui, J. Smith, K. Taylor, O. Wroe Wright, H. K. Baryan, G. Boyd, H. Conchie, L. Cox, J. Davies, S. Gardner, N. Hill, K. Krishna, F. Lakin, S. Scotcher, J. Alberts, M. Asad, J. Barraclough, A. Campbell, D. Marshall, W. Wakeford, P. Cronbach, F. D’Souza, E. Gammeri, J. Houlton, M. Hall, A. Kethees, R. Patel, M. Perera, J. Prowle, M. Shaid, E. Webb, S. Beattie, M. Chadwick, O. El-Taji, S. Haddad, M. Mann, M. Patel, K. Popat, L. Rimmer, H. Riyat, H. Smith, C. Anandarajah, M. Cipparrone, K. Desai, C. Gao, E. T. Goh, M. Howlader, N. Jeffreys, A. Karmarkar, G. Mathew, H. Mukhtar, E. Ozcan, A. Renukanthan, N. Sarens, C. Sinha, A. Woolley, R. Bogle, O. Komolafe, F. Loo, D. Waugh, R. Zeng, A. Crewe, J. Mathias, A. Mills, A. Owen, A. Prior, I. Saunders, A. Baker, L. Crilly, J. McKeon, H. K. Ubhi, A. Adeogun, R. Carr, C. Davison, S. Devalia, A. Hayat, R. B. Karsan, C. Osborne, K. Scott, C. Weegenaar, M. Wijeyaratne, F. Babatunde, E. Barnor-Ahiaku, G. Beattie, P. Chitsabesan, O. Dixon, N. Hall, N. Ilenkovan, T. Mackrell, N. Nithianandasivam, J. Orr, F. Palazzo, M. Saad, L. Sandland-Taylor, J. Sherlock, T. Ashdown, S. Chandler, T. Garsaa, J. Lloyd, S. Y. Loh, S. Ng, C. Perkins, A. Powell-Chandler, F. Smith, R. Underhill, N. Goergen, A. McKinley, C. Neary, N. Rafiq, A. Badran, N. Fearnhead, M. Leadon, M. Yin Lin Ting, K. Conlon, D. Ganesan, D. O’Connor, M. J. Arthur, Z. Panayi, S. Rehman, H. Awni, R. Rao, A. Robinson, J. Baxter, P. Loughlin, A. Ahmed, H. Barrow, M. T. Liviu, G. Harinath, S. Raveendran, S. Sait, A. Ali, M. Latter, S. Udalov, M. Bergstrom, H. Tabry, E. West, S. Dindyal, C. Gao, H. Patel, M. Bath, K. Bevan, M. Bica, X. M. Chan, J. Lee, S. O’Donnell, M. Ravindran, E. Blessing, J. H. De Sousa Magalhaes, P. Jain, B. Campbell, R. Evans, S. Poo, C. Sanghera, N. Standfield, D. Karponis, A. Mehdi, R. Patel, J. O’Callaghan, A. Kumar, M. Saat, S. Davidson, A. Hylands, E. McKie, R. Hughes, J. Latter, E. Leung, P. Dos Santos Jorge, J. Saramunda, S. Vig, P. Serebriakoff, J. Wayman, S. K. Yen, M. Coleman, S. Leong, I. Sajid, T. Tolppa, A. Fawole, D. Kandola, A. Khan, F. Babatunde, A. Harlinska, K. Sasapu, A. D. Durham-Hall, G. Fowler, M. Glithero, T. Stringfellow, A. Tulloch, A. Bagchi, A. Grant, O. Onibere, M. Bews-Hair, N. Rajaraman, T. Agarwal, S. Rabinowicz, A. Radford, E. Pedlar, A. Raja, H. Rshaidat, P. Y. A. Aw, E. MacKle, E. Y. L. Yap, R. Charnley, L. A. M. Lim, M. Naylor, B. Stainer, N. Alseed, R. Amarasinghe, R. Rajagopal, P. Horgan, S. Sohrabi, A. Wilkinson, N. Liew, J. Smallwood, N. Walker, E. Mutengesa, T. Rankin, K. Waite, E. Robertson-Waters, S. Stanger, C. Thorn, A. Botha, A. Fowler, T. Suri, P. Vickers, S. Denley, W. Johnston, L. Jiao, A. Pain, K. Vutipongsatorn, A. Kale, R. S. Karri, K. Waite, C. Johnson, J. Smith, C. Walsh, N. Dewan, J. Prowle, K. Theodoropoulou, P. Jain, T. Nisar, A. Ali, L. Chung, J. Thomas, M. Abbas, S. Mookerjee, J. Pitt, E. Budd, T. Fung, M. Li, D. MacAfee, N. Havers, A. Kelkar, M. Hanson, R. Ingleton, N. Sukirthan, A. Chang, I. Eiben, M. Qamar, H. Javanmard, N. Watson, D. Bahadori, I. Bloom, G. Pike, J. Black, M. Metcalfe, A. Radhakrishnan, J. Seehra, K. Almeida, H. Amin, R. Holdsworth, J. Yeung, S. Gurjar, R. Jones, M. Patel, A. Alam, H. Ali, J. BannardSmith, R. Khaw, A. Rais, R. Ahluwalia, E. Briggs, H. Gil, J. Clements, R. Cowden, L. McCarthy, S. Chan, S. F. Hussain, R. Hryniv, H. Noble, J. Olivier, J. Prowle, S. Sait, E. Elseedawy, A. Hassane, I. Ibrahim, T. Melaugh, A. Ali, L. Ashraf, S. Green, K. Chapple, E. Heywood, N. Ngonyamo, I. Nyamali, A. Patil, T. Bamford, O. Fafemi, C. Grieco, K. Khan, A. Martin, H. Seehra, A. Burke-Smith, N. Johnson, G. Samarth, K. Sun, J. Warusavitarne, S. Green, C. Maxwell-Armstrong, J. Sivaraj, A. Campbell, M. Elseedawy, E. Elseedawy, O. Kouli, S. Bradbury, R. Dennis, H. Walji, J. Hale, P. Haray, P. Eiben, A. Light, T. Singhal, N. Carter, F. Ewbank, C. Perrott, I. Chappelow, R. Hashemi, A. Lee, J. Matthews, T. Pinkney, M. Byrne, H. Eltyeb, P. O’Loughlin, C. Donaldson, O. Oke, K. Bisset, P. Glen, S. Norman, L. Tan, M. Ahmed, C. Maxwell-Armstrong, S. Rangarajan, J. Sivaraj, C. Hancock, S. Moug, S. Smith, G. Nowell, B. Rigney, A. Widdinson, C. Boereboom, J. Lund, W. Simpson, J. Wright, I. Fitzgerald, S. Mansfield, E. Shakweh, K. Whitehurst, Z. Lee, B. Pinnell, G. Williams, R. Broll, T. Drake, E. Harrison, C. McCann, T. Abbott, S. Mahdi, F. Nawab, J. Prowle, B. Butcher, P. D. Loganathan, L. A. Paterson, K. Pursnani, J. Atley, K. Hamdan, E. Mills, B. Clements, G. Donaldson, L. Eaton, R. Aftab, M. Gough, B. Griffiths, C. Ng, G. Nolan, J. Archer, V. Do, S. Sharma, J. Epstein, P. Sodde, B. J. Storey, H. Ahmad, N. Akram, T. Sami, F. Sheldon, H. Croft, L. Han, K. Lasithiotakis, J. Acharya, O. Adeleye, G. Kaur, N. Dabab, P. Kangesu, J. Knight, K. Srikathrikamanathan, H. Wilson, E. Dell, L. Ellis, K. McDonald, D. Sobhanpanah, K. Foster, J. Mogg, S. Subramonia, P. Hill, A. Rahem, F. Reid, R. Bachar, N. Greenough, L. Hlukha, A. Ramwell, S. Carlton-Carew, M. Murray, A. Raja, D. Burke, M. El-Haddad, L. B. Mecia, N. Patel, R. Bhatt, W. J. Koay, L. Y. H. Low, J. Reynolds, S. Abbott, H. Devan Nair, J. J. Lee, R. O’Connell, W. Carr, S. Davies, S. Unsworth, J. Ashcroft, D. Lazenby, D. Subar, S. Choi, S. Rinkoff, N. Sarens, M. Varcada, N. Ellerby, A. Hammad, N. McCartan, U. Muhammad, M. Howlader, E. Norman, P. Polly, S. Brown, T. Clark, N. Thakral, P. Hann, R. Henderson, S. Kirk, S. Gupta, T. Richards, J. Ting, M. Byrne, C. Byrne, J. Cheema, S. Walsh, C. Borg, J. Hardie, Y. Sardar, B. Hughes, S. Saeed, F. Saeed, A. Sharma, E. Ang, B. Kansu, M. Stechman, R. Walford, C. Woodward, S. Adeyemi, R. Awad, L. Imam, I. Leptidis, E. D. Kennedy, H. Patterson, Z. M. Soh, L. Walls, J. D. Yau, B. Ali, D. Evans, J. Smith, E. James, V. E. Kantola, K. Krishna, H. Naeem, J. Prowle, O. Komolafe, E. Tilling, C. Osborne, J. Schuster Bruce, C. Weegenaar, P. Chitsabesan, A. Goaman, C. Goode, N. Nithianandavisam, Dmitri Nepogodiev

**Affiliations:** Academic Department of Surgery, Heritage Building, University of Birmingham, Birmingham, UK

**Keywords:** Research collaborative, Surgery, Follow-up, Methodology

## Abstract

**Background:**

Trainee research collaboratives (TRCs) have pioneered high quality, prospective ‘snap-shot’ surgical cohort studies in the UK. Outcomes After Kidney injury in Surgery (OAKS) was the first TRC cohort study to attempt to collect one-year follow-up data. The aims of this study were to evaluate one-year follow-up and data completion rates, and to identify factors associated with improved follow-up rates.

**Methods:**

In this multicentre study, patients undergoing major gastrointestinal surgery were prospectively identified and followed up at one-year following surgery for six clinical outcomes. The primary outcome for this report was the follow-up rate for mortality at 1 year. The secondary outcome was the data completeness rate in those patients who were followed-up. An electronic survey was disseminated to investigators to identify strategies associated with improved follow-up.

**Results:**

Of the 173 centres that collected baseline data, 126 centres registered to participate in one-year follow-up. Overall 62.3% (3482/5585) of patients were followed-up at 1 year; in centres registered to collect one-year outcomes, the follow-up rate was 82.6% (3482/4213). There were no differences in sex, comorbidity, operative urgency, or 7-day postoperative AKI rate between patients who were lost to follow-up and those who were successfully followed-up. In centres registered to collect one-year follow-up outcomes, overall data completeness was 83.1%, with 57.9% (73/126) of centres having ≥95% data completeness. Factors associated with increased likelihood of achieving ≥95% data completeness were total number of patients to be followed-up (77.4% in centres with < 15 patients, 59.0% with 15–29 patients, 51.4% with 30–59 patients, and 36.8% with > 60 patients, *p* = 0.030), and central versus local storage of patient identifiers (72.5% vs 48.0%, respectively, *p* = 0.006).

**Conclusions:**

TRC methodology can be used to follow-up patients identified in prospective cohort studies at one-year. Follow-up rates are maximized by central storage of patient identifiers.

## Background

Trainee research collaboratives (TRCs) have pioneered methods of rapidly delivering high quality, prospective, cross-sectional ‘snapshots’ of surgical practice and outcomes [1, 2]. TRC studies are led by frontline clinicians and students, without the need for significant additional infrastructural resources or funding. They capture data over short time periods across multiple centres, collating large datasets [3–5] that can be used to generate hypotheses for future randomised trials and identify targets for national quality improvement [6–10].

TRC studies are delivered by student and postgraduate trainees who rotate between hospitals at least once every 12 months, which would create discontinuity within local teams if studies were run over protracted periods of time. Consequently, most surgical TRC studies follow patients up to the point of discharge or to postoperative day 30; no published observational studies from TRCs have undertaken outcome assessment beyond 6 months [6–10]. In planning longer term follow-up, a particular challenge is ensuring safe local storage of patient identifiers so that patients can be followed-up at one-year even if the original study collaborators at that site have rotated to continue their training at another centre.

Outcomes After Kidney injury in Surgery (OAKS) was the first TRC cohort study to attempt to collect to one-year follow-up data. The aim of this study was to evaluate one-year follow-up and data completion rates, and to identify factors associated with improved follow-up rates.

## Methods

### Student Audit and Research in Surgery (STARSurg)

Student Audit and Research in Surgery (STARSurg) is the UK’s national medical student research collaborative. It is coordinated by a team of medical students and postgraduate trainees. The collaborative model and the educational benefits to participating students have been described previously [11, 12]. STARSurg studies are delivered by ‘mini-teams’ at each centre consisting of consultant surgeons, junior doctors and medical students.

### Outcomes After Kidney injury in Surgery

Outcomes After Kidney injury in Surgery (OAKS) [13] is a multicentre study which prospectively identified patients in the UK and Republic of Ireland undergoing major gastrointestinal or liver resection, or reversal of ileostomy or colostomy from 23 September 2015 to 18 November 2015. In the United Kingdom, the South-East Scotland Research Ethics Service (reference: NR/1506AB4) confirmed that ethical review was not required, as this observational study only collected routine, non- patient identifiable data. Individual participating UK centres were responsible for registering the study locally as either clinical audit or service evaluation. In the Republic of Ireland, participating centres were responsible for securing research ethics approval locally, as required by institutional regulations. The 30-day outcomes from the OAKS study have been reported previously [14, 15].

Data was collected on the Research Electronic Data Capture (REDCap) system, an online platform for secure web-based data collection. The REDCap platform was developed in 2004 at Vanderbilt University, which is a secure data collection tool meeting the Health Insurance Portability and Accountability Act (HIPAA) compliance standards. Patients’ hospital or NHS identification numbers and linked study-specific identification numbers were stored in accordance with local Caldicott Guardian approvals; either centrally on the REDCap system or within an encrypted spreadsheet held securely on the local hospital computer network by a member of the data collection team (a local investigator, supervising consultant, or audit officer).

In the period November 2016 to May 2017 the STARSurg network collected one-year outcomes for the patients identified in the initial prospective patient enrolment phase of OAKS. Patients were excluded from one-year follow-up if they had died within 30 days of index surgery, as there would be no additional data to collect from these patients since the 30-day follow-up that had already been completed previously. At centres that had participated in initial OAKS data collection, new mini-teams were recruited to complete one-year follow-up. The clinical endpoints collected at one-year were (1) mortality at 1-year, (2) myocardial infarction or cerebrovascular accident at 1-year, (3) total combined hospital length of stay up to 1-year postoperatively, (4) the most recent available serum creatinine value up to 1-year, (5) nephrology review at 1-year, and (6) dialysis at 1-year. These clinical endpoints based on a review of the literature on postoperative AKI [16–19]. In this observational study, clinic follow-up visits and blood tests were arranged by clinical teams according to their normal practice. No additional follow-up visits or blood tests were arranged for this study. Follow-up was considered to have been achieved if patients’ records had been successfully reviewed, even if no creatinine tests had been completed by the clinical team during the follow-up period.

Centres were considered to have registered for collection of one-year follow-up if a data collection mini-team was established at the site, institutional approval was granted for collected of follow-up data, and at least one patient was followed-up at the site.

### Outcome measures

The primary outcome for this report was the mortality follow-up rate for mortality. This was the proportion of patients for whom the primary endpoint (mortality) was followed-up at 1-year. The secondary outcome was the data completeness rate in centres that registered to collect one-year follow-up. The data completion rate was the proportion of patients with complete data for all six clinical endpoints.

### Investigator feedback survey

Following locking of the OAKS database, an electronic survey was disseminated to all investigators who had participated in one-year follow-up (Additional file 1: Table S1). This assessed investigators’ experience of one-year follow-up data collection. 5-point Likert scales were used to assess investigators’ experience of the following (from 1 = very difficult, to 5 = very easy): identifying a supervising consultant; registering the audit; linking patient hospital identifier to the study-specific identifier; collecting data using local hospital computer systems, or paper records. For analysis, scores of 4 to 5 out of 5 were categorised as “Positive”, and scores of 1 to 3 out of 5 were categorised as “Negative” responses to create a dichotomous variable.

### Statistical analysis

The baseline characteristics of patients lost to follow-up were compared to those patients who were successfully followed-up. Continuous variables were expressed as mean with standard deviation, or median with interquartile range. Continuous variables were analysed using t-test or Mann-Whitney test, where appropriate. Categorical variables were expressed as percentages and analysed using Chi-squared test, or with Fisher’s exact modification if expected cell counts were less than five. For all analyses, a *p*-value of < 0.05 was considered as statistically significant. Data analysis was undertaken using R Foundation Statistical Software (R 3.2.1, R Foundation for Statistical Computing, Vienna, Austria).

## Results

### Centre registration

**Fig. 1:**
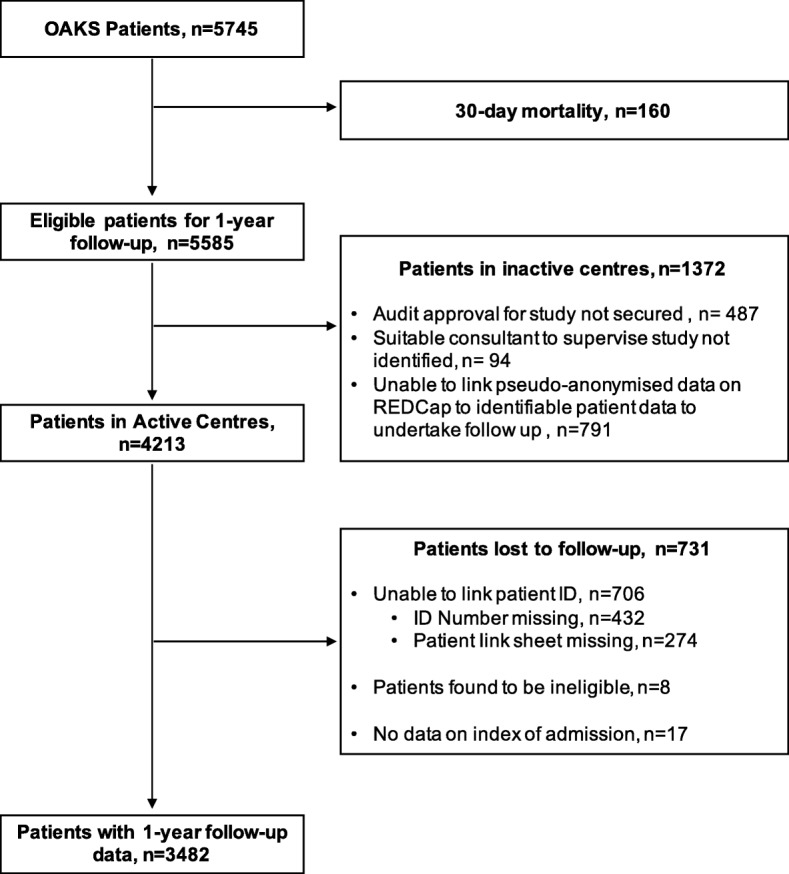
Flowchart of 1-year follow-up in the OAKS study

Of 173 centres that had collected baseline data in the initial phase of OAKS, 126 centres registered to participate in one-year follow-up. Of the 47 centres that did not register, 35 were unable to obtain patient ID link sheets, and 12 were not granted audit and/or Caldicott Guardian approval prior to the data collection deadline (Fig. 1). Centres in Scotland, Ireland, and England all achieved similar levels of registration to participate in one-year follow-up (88.9% vs 78.6% vs 72.5% respectively, Table 2), however there were significantly fewer centres in Wales (40.0%). Centres at which a junior doctor was engaged in the process were more likely to register to enter data (80.6% vs 46.2%, *p* < 0.001). Centres which had stored patient hospital identifiers on the central REDCap system during the initial data collection phase had a significantly higher participation rate in one-year follow-up (83.6% vs 67.0%, *p* = 0.019). However, prior centre participation in STARSurg projects preceding OAKS did not affect the likelihood of centres registering to collect one-year follow-up (74.8% vs 61.5%, *p* = 0.160).

### Follow-up rates

The initial data collection phase of OAKS captured 5745 patients, of whom 5585 remained alive at 30-days following their index procedure (Fig. 1) and eligible for one-year follow-up. Overall 62.3% (3482/5585) of patients were followed-up at 1 year. Of the 2103 patients lost to follow-up, 65.2% (1372) were from the 47 centres that did not register to participate in one-year follow-up. In registered centres, the follow-up rate was 82.6% (3482/4213).

### Characteristics of patients followed-up at one-year

There were no significant differences in age, American Society of Anaesthesiologists (ASA) grade, Revised Cardiac Risk Index (RCRI), urgency of surgery and contamination between patients with and without follow-up at one-year (Table 1). However, patients who were followed-up had significantly higher rates of open surgery compared to patients that were not followed-up (61.5% vs 52.0%, *p* < 0.001). There were no significant difference in rates of AKI (12.7% vs 10.7%, *p* = 0.060) between patients with and without one-year follow-up.

**Table 1:** Characteristics of patients with one-year follow-up completed

		No follow-up (*n* = 2104)	Follow-up (*n* = 3481)	*p*-value
Age (years)	Mean (SD)	62.8 (15.9)	62.6 (16)	0.689
Gender	Female	1561 (44.8)	967 (46.0)	0.417
	Male	1920 (55.2)	1137 (54.0)	
ASA Grade	ASA I-II	2176 (67.0)	1312 (68.5)	0.279
	ASA III-V	1071 (33.0)	604 (31.5)	
Operative urgency	Elective	2752 (79.1)	1687 (80.2)	0.314
	Emergency	729 (20.9)	417 (19.8)	
RCRI score	< 3	3328 (95.7)	2014 (96.0)	0.491
	≥3	151 (4.3)	83 (4.0)	
Operative approach	Laparoscopic	1339 (38.5)	1006 (48.0)	< 0.001
	Open/ laparoscopic converted to open	2136 (61.5)	1090 (52.0)	
Operative contamination	Clean-contaminated	3234 (93.2)	1928 (91.9)	0.065
	Contaminated	235 (6.8)	170 (8.1)	
AKI within 7 days of index surgery	No AKI	2979 (85.6)	1837 (87.3)	0.060
	AKI	443 (12.7)	225 (10.7)	
	Missing	59 (1.7)	42 (2.0)	

*AKI* Acute Kidney Injury, *ASA* American Society of Anaesthesiologists, *RCRI* Revised Cardiac Risk Index

### Data completeness

Centre characteristics associated with ≥95% completeness are presented in Table 2. In centres registered to collect 1-year follow-up outcomes, overall data completeness was 83.1%. Of the 126 of centres that participated, 57.9% (*n* = 73) had ≥95% data completeness. Scotland had significantly more centres with ≥95% data completeness (100.0%) compared to England, Ireland, and Wales (55.8% vs 36.4% vs 0%, *p* < 0.001). The more patients a centre had to follow-up, the less likely it was to achieve ≥95% data completeness (< 15: 77.4% vs 15–29: 59.0% vs 30–59: 51.4 vs > 60: 36.8%). Centres storing patient identifiers on the central REDCap system had significantly higher rates of ≥95% data completeness than those storing identifiers locally (72.5% vs 48.0%, respectively, *p* < 0.001).

**Table 2:** OAKS centre characteristics, centre activity and data completeness at one-year postoperatively

		Centre active in OAKS			Centre ≥95% completeness in OAKS		
		Active (*n* = 126)	Inactive (*n* = 47)	*p*-value	Yes (n = 73)	No (*n* = 53)	*p*-value
UK Countries	England	95 (72.5)	36 (27.5)	0.045	53 (55.8)	42 (44.2)	< 0.001
	Ireland	11 (78.6)	3 (21.4)		4 (36.4)	7 (63.6)	
	Scotland	16 (88.9)	2 (11.1)		16 (100.0)	0 (0.0)	
	Wales	4 (40.0)	6 (60.0)		0 (0.0)	4 (100.0)	
Total number of patients to be followed up	< 15 patients	31 (70.5)	13 (29.5)	0.491	24 (77.4)	7 (22.6)	0.030
	15–29 patients	39 (72.2)	15 (27.8)		23 (59.0)	16 (41.0)	
	30–59 patients	37 (69.8)	16 (30.2)		19 (51.4)	18 (48.6)	
	> 60 patients	19 (86.4)	3 (13.6)		7 (36.8)	12 (63.2)	
Percentage of patients with complete follow-up	Mean (SD)	–	–	–	28.3 (21.6)	27.9 (21.9)	0.877
Junior doctor present in OAKS mini-team^a^	Yes	108 (80.6)	26 (19.4)	< 0.001	63 (58.3)	45 (41.7)	0.825
	No	18 (46.2)	21 (53.8)		10 (55.6)	8 (44.4)	
Central storage of patient hospital identifiers	Yes	51 (83.6)	10 (16.4)	0.019	37 (72.5)	14 (27.5)	0.006
	No	75 (67.0)	37 (33.0)		36 (48.0)	39 (52.0)	
Survey respondents	Yes	125 (84.5)	23 (15.5)	< 0.001	72 (57.6)	53 (42.4)	0.392
	No	1 (4.0)	24 (96.0)		1 (100.0)	0 (0.0)	

^a^*Junior doctors are present in each mini-team over a data collection period*

### Investigator feedback survey

Survey responses were received from 285 students and junior doctors, a 78% (285/365) response rate. At least one response was received from 86% (148/173) of centres that participated in initial data collection in 2015. Of centres that returned the survey, 59 (40.0%) had 100% data completion of one-year follow-up, 72 (48.6%) had ≥95% data completion and 23 (15.5%) did not register to submit one-year follow-up data. Table 3 summarises respondent characteristics and experience of OAKS-2 by data completeness. Only collaborators with positive experience of linking patient ID were more likely to achieve > 95% data completeness (71.6% vs 37.6%, *p* < 0.001). There was no association between perceived difficulty with registering audit, data collection, and identifying a supervising consultant, and > 95% data completeness. Following this study, a summary of recommendations for future multi-centre collaborative studies with longitudinal follow-up were developed and presented in Table 4.

**Table 3:** OAKS collaborator survey responses, centre activity and data completeness at one-year postoperatively

		Respondent at a centre with ≥95% completeness		
		Yes (*n* = 141)	No (*n* = 111)	*p*-value
Stage of Training	Junior Doctor	47 (56.6)	36 (43.4)	0.929
	Later Year Student	52 (56.5)	40 (43.5)	
	Early Year Student	41 (53.9)	35 (46.1)	
Previous participation in initial phase of OAKS data collection	Yes	66 (56.4)	51 (43.6)	0.850
	No	74 (55.2)	60 (44.8)	
Prior experience with audit	Yes	65 (60.2)	43 (39.8)	0.222
	No	75 (52.4)	68 (47.6)	
Rating of experience identifying consultant	Positive (4–5)	91 (58.7)	64 (41.3)	0.235
	Not Positive (< 4)	49 (51.0)	47 (49.0)	
Rating of experience registering audit ^a^	Positive (4–5)	67 (56.8)	51 (43.2)	0.763
	Not Positive (< 4)	73 (54.9)	60 (45.1)	
Rating of experience linking Patient ID ^a^	Positive (4–5)	96 (71.6)	38 (28.4)	< 0.001
	Not Positive (< 4)	44 (37.6)	73 (62.4)	
Rating of experience collecting data ^a^	Positive (4–5)	106 (59.2)	73 (40.8)	0.104
	Not Positive (< 4)	34 (47.9)	37 (52.1)	

^a^Rated on a self-reported Likert scale between 1 (very difficult) and 5 (very easy)

**Table 4:** Summary of recommendations for future multi-centre collaborative studies with longitudinal follow-up

Number	Recommendation to improve completeness of longitudinal follow-up
1. Study Design Recommendations	
1.1	Linked patient identifiers should be kept in a central repository (for example a REDCap system) if Caldicott Guardian approval is given to minimise loss to follow-up
2. Study Delivery Recommendations	
2.1	Included at least one team member with previous experience in trainee research collaborative projects in each data collection team, where possible.
2.2	Having junior doctors paired to students in data collection teams improve centre participation and data completeness rates. Where this is not possible, at least one senior medical student with previous collaborative audit experience should be part of the data collection team.
2.3	A network of regional leads are useful to monitor local progress and feedback to steering committee
2.4	Tracking regional variation in performance through the study and targeting specific efforts to improve follow-up and data completeness in these areas may increase data quality and maximise efficiency.
2.5	In high-volume centres where achieving high data completeness may be burdensome, consider permitting involvement additional team members to provide support.

## Discussion

OAKS was the first TRC prospective cohort study to attempt to complete longitudinal one-year follow-up. This report demonstrates that most centres were able to collect one-year follow-up data with high levels of data completeness. Although the overall follow-up rate was only 62%, there was no evidence of systematic bias in patients being followed-up. Factors associated with increased likelihood of achieving > 95% data completeness were lower numbers of patients to be followed-up, and central storage of patient hospital identifiers. As TRCs have now been set up across Europe [20, 21], validating this methodology will have broad international benefits.

Most studies that complete longitudinal follow-up of prospectively identified patients [22] require patient consent, ethical approval, and significant research infrastructural funding. Even in well resourced, funded trials loss to follow-up of up to 15% is expected and built in to sample size calculations [23]. In the UK, National Research Ethics Service regulations permitted collection of one-year outcomes to be completed as clinical audit, without the need for research ethics approval. Without ethical approval it was not possible to collect identifiable data centrally. This report demonstrates that satisfactory follow-up is achievable within this regulatory framework, and without dedicated funding.

The most commonly reported barrier to achieving one-year follow-up was inability to identify linked patient records. Methods for maintaining linkage between hospital identifiers and study-specific identifiers were either holding hospital identifiers directly on the REDCap system or holding a cross-reference of hospital and study-specific identifiers on hospital computer systems by audit offices or consultants. Collaborating investigators found it easier to complete data collection when approved hospital identifiers were stored on the REDCap system. In Scotland, where national approval was gained for Community Health Index (CHI) identifiers to be stored on REDCap, data completion rates were higher. Therefore, future studies should seek local or national Caldicott guardian approval to store approved hospital identifiers on REDCap.

Loss to follow-up presents a major risk to the internal validity of a study as it leaves a specific population where outcomes remain unassessed, which may differ between groups. In the OAKS study, there were no significant differences in the patient-level demographics, operative indications or ASA grades between the group that underwent one-year follow-up and those that did not. The AKI and mortality rates at 30-days postoperative follow-up also were not significantly different between the groups that did and did not achieve one-year follow-up data.

A significant limitation to the method of follow-up in this study was its restriction to the hospital where the index surgery was performed. A small number of patients may choose to move their care to another centre, and some patients may be readmitted to a different hospital. Consequently, when patients were followed-up at the index hospital, there may have been no record of readmissions, treatments, and blood tests that took place at other hospitals. In addition, since no specific clinic visits were arranged for this study, if clinical teams did not arrange any postoperative clinic visits, or patients did not attend arranged visits, it is possible that the hospital records that were reviewed as source data for this study may not have been fully accurate.

The evaluation of this study’s methodology was limited by the broadness of the barriers explored in the investigator survey. A qualitative approach with detailed interviews with investigators may have been more likely to identify specific difficulties that precluded follow-up from being completed. Incorporating such a qualitative component to future studies may improve follow-up by identifying more solutions [24].

## Conclusion

The OAKS study has demonstrated that prospective TRC cohort studies can successfully complete one-year longitudinal follow-up, with acceptable data completeness rates. Future studies may maximise follow-up rates by optimising procedures for storage of patient identifiers, embedding collaborators with previous experience of TRC studies within data collection team, and tracking regional variation in performance throughout the study.

## Supplementary information

**Additional file 1: Table S1.** OAKS collaborator survey responses, centre activity and data completeness at one-year postoperatively.

## Data Availability

The datasets used and/or analysed during the current study are available from the corresponding author on reasonable request.

## Abbreviations

AKI: Acute Kidney Injury
ASA: American Society of Anaesthesiologists
CHI: Community Health Index
OAKS: Outcomes After Kidney injury in Surgery
RCRI: Revised Cardiac Risk Index
REDCap: Research Electronic Data Capture
STARSurg: *Student Audit and Research in Surgery*
TRCs: Trainee research collaboratives
