# Correction to: Challenges of one-year longitudinal follow-up of a prospective, observational cohort study using an anonymised database: recommendations for trainee research collaboratives

**DOI:** 10.1186/s12874-020-0909-3

**Published:** 2020-02-07

**Authors:** STARSurg Collaborative, Sivesh Kamarajah, Kenneth A. McLean, Aditya Borakati, Thomas M. Drake, Evelina Woin, Chetan Khatri, J. Edward Fitzgerald, Ewen M. Harrison, Aneel Bhangu, Dmitri Nepogodiev, James C. Glasbey, Joshua Burke, Michael F. Bath, Henry A. Claireaux, Buket Gundogan, Midhun Mohan, Praveena Deekonda, Chia Kong, Holly Joyce, Lisa Mcnamee, Nishkantha Arulkumaran, Samira Bell, Fiona Duthie, Jeremy Hughes, Thomas D. Pinkney, John Prowle, Toby Richards, Mark Thomas, K. Dynes, M. Patel, P. Patel, C. Wigley, R. Suresh, A. Shaw, S. Klimach, P. Jull, D. Evans, R. Preece, I. Ibrahim, V. Manikavasagar, R. Smith, F. S. Brown, P. Deekonda, R. Teo, D. P. Y. Sim, A. Borakati, A. E. Logan, I. Barai, H. Amin, S. Suresh, R. Sethi, W. Gul, W. Bolton, O. Corbridge, L. Horne, M. Attalla, R. Morley, C. Robinson, T. Hoskins, R. McAllister, S. Lee, Y. Dennis, G. Nixon, E. Heywood, H. Wilson, L. Ng, S. Samaraweera, A. Mills, C. Doherty, E. Woin, J. Belchos, V. Phan, M. Arnold, S. Sheik-Ali, R. Suresh, A. Cordaro, E. Mills, N. Lorch, D. Thomas, B. Ibrahim, S. Chee, T. Ngan, S. Pronin, N. Thakral, T. Yeoh, J. Wilson, R. Goodson, P. Molloy, M. Akhbari, W. Gul, R. Helliwell, S. Rees, M. Al-Attar, N. Griffiths, J. Mayes, P. Thomas, S. George, A. Thind, M. Kerr, F. Shafiq, I. Yasin, M. Gallagher, E. Sewart, T. Chouari, T. Gardner, N. Goergen, J. D. B. Hayes, C. S. MacLeod, R. McCormack, A. McKinley, S. McKinstry, W. Milligan, L. Ooi, N. M. Rafiq, T. Sammut, E. Sinclair, M. Smith, C. Baker, A. P. R. Boulton, J. Collins, H. C. Copley, N. Fearnhead, H. Fox, T. Mah, J. McKenna, V. Naruka, N. Nigam, B. Nourallah, S. Perera, A. Qureshi, S. Saggar, L. Sun, X. Wang, D. D. Yang, P. Caroll, C. Doyle, S. Elangovan, A. Falamarzi, K. Gascon Perai, E. Greenan, D. Jain, M. Lang-Orsini, S. Lim, L. O’Byrne, P. Ridgway, S. Van der Laan, J. Wong, J. Arthur, J. Barclay, P. Bradley, C. Edwin, E. Finch, E. Hayashi, M. Hopkins, D. Kelly, M. Kelly, N. McCartan, A. Ormrod, A. Pakenham, J. Hayward, C. Hitchen, A. Kishore, T. Martins, J. Philomen, R. Rao, C. Rickards, N. Burns, M. Copeland, C. Durand, A. Dyal, A. Ghaffar, A. Gidwani, M. Grant, C. Gribbon, A. Gruhn, M. Leer, K. Ahmad, G. Beattie, M. Beatty, G. Campbell, G. Donaldson, S. Graham, D. Holmes, S. Kanabar, H. Liu, C. McCann, R. Stewart, S. Vara, O. Ajibola-Taylor, E. J. E. Andah, C. Ani, N. M. O. Cabdi, G. Ito, M. Jones, A. Komoriyama, P. Patel, L. Titu, M. Basra, P. Gallogly, G. Harinath, S. H. Leong, A. Pradhan, I. Siddiqui, S. Zaat, A. Ali, M. Galea, W. L. Looi, J. C. K. Ng, G. Atkin, A. Azizi, Z. Cargill, Z. China, J. Elliot, R. Jebakumar, J. Lam, G. Mudalige, C. Onyerindu, M. Renju, V. Shankar Babu, M. Hussain, N. Joji, B. Lovett, H. Mownah, B. Ali, B. Cresswell, A. K. Dhillon, Y. S. Dupaguntla, C. Hungwe, J. D. Lowe-Zinola, J. C. H. Tsang, K. Bevan, C. Cardus, A. Duggal, S. Hossain, M. McHugh, M. Scott, F. Chan, R. Evans, E. Gurung, B. Haughey, B. Jacob-Ramsdale, M. Kerr, J. Lee, E. McCann, K. O’Boyle, N. Reid, F. Hayat, S. Hodgson, R. Johnston, W. Jones, M. Khan, T. Linn, S. Long, P. Seetharam, S. Shaman, B. Smart, A. Anilkumar, J. Davies, J. Griffith, B. Hughes, Y. Islam, D. Kidanu, N. Mushaini, I. Qamar, H. Robinson, M. Schramm, C. Yan Tan, H. Apperley, C. Billyard, J. M. Blazeby, S. P. Cannon, S. Carse, A. Göpfert, A. Loizidou, J. Parkin, E. Sanders, S. Sharma, G. Slade, R. Telfer, I. Whybrow Huppatz, E. Worley, L. Chandramoorthy, C. Friend, L. Harris, P. Jain, M. J. Karim, K. Killington, J. McGillicuddy, C. Rafferty, N. Rahunathan, T. Rayne, Y. Varathan, N. Verma, D. Zanichelli, M. Arneill, F. Brown, B. Campbell, L. Crozier, J. Henry, C. McCusker, P. Prabakaran, R. Wilson, U. Asif, M. Connor, S. Dindyal, N. Math, A. Pagarkar, H. Saleem, I. Seth, S. Sharma, N. Standfield, T. Swartbol, R. Adamson, J. E. Choi, O. El Tokhy, W. Ho, N. R. Javaid, M. Kelly, A. S. Mehdi, D. Menon, I. Plumptre, S. Sturrock, J. Turner, O. Warren, E. Crane, B. Ferris, C. Gadsby, J. Smallwood, M. Vipond, V. Wilson, T. Amarnath, A. Doshi, C. Gregory, K. Kandiah, B. Powell, H. Spoor, C. Toh, R. Vizor, M. Common, K. Dunleavy, S. Harris, C. Luo, Z. Mesbah, A. Prem Kumar, A. Redmond, S. Skulsky, T. Walsh, D. Daly, L. Deery, E. Epanomeritakis, M. Harty, D. Kane, K. Khan, R. Mackey, J. McConville, K. McGinnity, G. Nixon, A. Ang, J. Y. Kee, E. Leung, S. Norman, S. V. Palaniappan, P. Partha Sarathy, T. Yeoh, J. Frost, P. Hazeldine, L. Jones, M. Karbowiak, C. Macdonald, A. Mutarambirwa, A. Omotade, M. Runkel, G. Ryan, N. Sawers, C. Searle, S. Suresh, S. Vig, A. Ahmad, R. McGartland, R. Sim, A. Song, J. Wayman, R. Brown, L. H. Chang, K. Concannon, C. Crilly, T. J. Arnold, A. Burgin, F. Cadden, C. H. Choy, M. Coleman, D. Lim, J. Luk, P. Mahankali-Rao, A. J. Prudence-Taylor, D. Ramakrishnan, J. Russell, A. Fawole, J. Gohil, B. Green, A. Hussain, L. McMenamin, L. McMenamin, M. Tang, F. Azmi, S. Benchetrit, T. Cope, A. Haque, A. Harlinska, R. Holdsworth, T. Ivo, J. Martin, T. Nisar, A. Patel, K. Sasapu, J. Trevett, G. Vernet, A. Aamir, C. Bird, A. Durham-Hall, W. Gibson, J. Hartley, N. May, V. Maynard, S. Johnson, C. Mc Donald Wood, M. O’Brien, J. Orbell, T. D. Stringfellow, F. Tenters, S. Tresidder, W. Cheung, A. Grant, N. Tod, M. Bews-Hair, Z. H. Lim, S. W. Lim, M. Vella-Baldacchino, S. Auckburally, A. Chopada, S. Easdon, R. Goodson, F. McCurdie, M. Narouz, A. Radford, E. Rea, O. Taylor, T. Yu, M. Alfa-Wali, L. Amani, I. Auluck, P. Bruce, J. Emberton, R. Kumar, N. Lagzouli, A. Mehta, A. Murtaza, M. Raja, I. S. Dennahy, K. Frew, A. Given, Y. Y. He, M. A. Karim, E. MacDonald, E. McDonald, D. McVinnie, S. K. Ng, A. Pettit, D. P. Y. Sim, S. D. Berthaume-Hawkins, R. Charnley, K. Fenton, D. Jones, C. Murphy, J. Q. Ng, R. Reehal, H. Robinson, S. S. Seraj, E. Shang, A. Tonks, P. White, A. Yeo, P. Chong, R. Gabriel, N. Patel, E. Richardson, L. Symons, D. Aubrey-Jones, S. Dawood, M. Dobrzynska, S. Faulkner, H. Griffiths, F. Mahmood, P. Patel, M. Perry, A. Power, R. Simpson, A. Ali, P. Brobbey, A. Burrows, P. Elder, R. Ganyani, C. Horseman, P. Hurst, H. Mann, K. Marimuthu, S. McBride, E. Pilsworth, N. Powers, P. Stanier, R. Innes, T. Kersey, M. Kopczynska, N. Langasco, N. Patel, R. Rajagopal, B. Atkins, W. Beasley, Z. Cheng Lim, A. Gill, H. Li Ang, H. Williams, T. Yogeswara, R. Carter, M. Fam, J. Fong, J. Latter, M. Long, S. Mackinnon, C. McKenzie, J. Osmanska, V. Raghuvir, A. Shafi, K. Tsang, L. Walker, K. Bountra, O. Coldicutt, D. Fletcher, S. Hudson, S. Iqbal, T. Lopez Bernal, J. W. B. Martin, F. Moss-Lawton, J. Smallwood, A. Cardwell, K. Edgerton, J. Laws, A. Rai, K. Robinson, K. Waite, J. Ward, H. Youssef, C. Knight, P. Y. Koo, A. Lazarou, S. Stanger, C. Thorn, M. C. Triniman, A. Botha, L. Boyles, S. Cumming, S. Deepak, A. Ezzat, A. J. Fowler, A. M. Gwozdz, S. F. Hussain, S. Khan, H. Li, B. Lu Morrell, J. Neville, R. Nitiahpapand, O. Pickering, H. Sagoo, E. Sharma, K. Welsh, S. Denley, S. Khan, M. Agarwal, N. Al-Saadi, R. Bhambra, A. Gupta, Z. A. R. Jawad, L. R. Jiao, K. Khan, G. Mahir, S. Singagireson, B. L. Thoms, B. Tseu, R. Wei, N. Yang, N. Britton, D. Leinhardt, M. Mahfooz, A. Palkhi, M. Price, S. Sheikh, M. Barker, D. Bowley, M. Cant, U. Datta, M. Farooqi, A. Lee, G. Morley, M. Naushad Amin, A. Parry, S. Patel, S. Strang, N. Yoganayagam, A. Adlan, S. Chandramoorthy, Y. Choudhary, K. Das, M. Feldman, B. France, R. Grace, H. Puddy, P. Soor, M. Ali, P. Dhillon, A. Faraj, L. Gerard, M. Glover, H. Imran, S. Kim, Y. Patrick, J. Peto, A. Prabhudesai, R. Smith, A. Tang, N. Vadgama, R. Dhaliwal, T. Ecclestone, A. Harris, D. Ong, D. Patel, C. Philp, E. Stewart, L. Wang, E. Wong, Y. Xu, T. Ashaye, T. Fozard, F. Galloway, S. Kaptanis, P. Mistry, T. Nguyen, F. Olagbaiye, M. Osman, Z. Philip, R. Rembacken, S. Tayeh, K. Theodoropoulou, A. Herman, J. Lau, A. Saha, M. Trotter, O. Adeleye, D. Cave, T. Gunwa, J. Magalhães, S. Makwana, R. Mason, M. Parish, H. Regan, P. Renwick, G. Roberts, D. Salekin, C. Sivakumar, A. Tariq, I. Liew, A. McDade, D. Stewart, M. Hague, N. Hudson-Peacock, C. E. S. Jackson, F. James, J. Pitt, E. Y. Walker, R. Aftab, J. J. Ang, S. Anwar, J. Battle, E. Budd, J. Chui, H. Crook, P. Davies, S. Easby, E. Hackney, B. Ho, S. Z. Imam, J. Rammell, H. Andrews, C. Perry, P. Schinle, P. Ahmed, T. Aquilina, E. Balai, M. Church, E. Cumber, A. Curtis, G. Davies, Y. Dennis, E. Dumann, S. Greenhalgh, P. Kim, S. King, K. H. M. Metcalfe, L. Passby, N. Redgrave, Z. Soonawalla, S. Waters, A. Zornoza, I. Gulzar, J. Hole, K. Hull, H. Ishaq, J. Karaj, A. Kelkar, E. Love, S. Patel, D. Thakrar, M. Vine, A. Waterman, N. P. Dib, N. Francis, M. Hanson, R. Ingleton, K. S. Sadanand, N. Sukirthan, S. Arnell, M. Ball, N. Bassam, G. Beghal, A. Chang, V. Dawe, A. George, T. Huq, A. Hussain, B. Ikram, L. Kanapeckaite, M. Khan, D. Ramjas, A. Rushd, S. Sait, M. Serry, E. Yardimci, S. Capella, L. Chenciner, C. Episkopos, E. Karam, C. McCarthy, W. Moore-Kelly, N. Watson, V. Ahluwalia, J. Barnfield, O. Ben-Gal, I. Bloom, A. Gharatya, K. Khodatars, N. Merchant, A. Moonan, M. Moore, K. Patel, H. Spiers, K. Sundaram, J. Turner, M. F. Bath, J. Black, H. Chadwick, L. Huisman, H. Ingram, S. Khan, L. Martin, M. Metcalfe, P. Sangal, J. Seehra, A. Thatcher, S. Venturini, I. Whitcroft, Z. Afzal, S. Brown, A. Gani, A. Gomaa, N. Hussein, S. Y. Oh, N. Pazhaniappan, E. Sharkey, T. Sivagnanasithiyar, C. Williams, J. Yeung, L. Cruddas, S. Gurjar, A. Pau, R. Prakash, R. Randhawa, L. Chen, I. Eiben, M. Naylor, D. Osei-Bordom, R. Trenear, J. Bannard-Smith, N. Griffiths, B. Y. Patel, F. Saeed, H. Abdikadir, M. Bennett, R. Church, S. E. Clements, J. Court, A. Delvi, J. Hubert, B. Macdonald, F. Mansour, R. R. Patel, R. Perris, S. Small, A. Betts, N. Brown, A. Chong, C. Croitoru, A. Grey, P. Hickland, C. Ho, D. Hollington, L. McKie, A. R. Nelson, H. Stewart, P. Eiben, M. Nedham, I. Ali, T. Brown, S. Cumming, C. Hunt, C. Joyner, C. McAlinden, J. Roberts, D. Rogers, A. Thachettu, N. Tyson, R. Vaughan, N. Verma, T. Yasin, K. Andrew, N. Bhamra, S. Leong, R. Mistry, H. Noble, F. Rashed, N. R. Walker, L. Watson, M. Worsfold, E. Yarham, H. Abdikadir, A. Arshad, B. Barmayehvar, L. Cato, N. Chan-lam, V. Do, A. Leong, Z. Sheikh, T. Zheleniakova, J. Coppel, S. T. Hussain, R. Mahmood, R. Nourzaie, J. Prowle, S. Sheik-Ali, A. Thomas, A. Alagappan, R. Ashour, H. Bains, J. Diamond, J. Gordon, B. Ibrahim, M. Khalil, D. Mittapalli, Y. N. Neo, P. Patil, F. S. Peck, N. Reza, I. Swan, M. Whyte, S. Chaudhry, J. Hernon, H. Khawar, J. O’Brien, M. Pullinger, K. Rothnie, S. Ujjal, S. Bhatte, J. Curtis, S. Green, A. Mayer, G. Watkinson, K. Chapple, T. Hawthorne, M. Khaliq, L. Majkowski, T. A. M. Malik, K. Mclauchlan, B. Ng Wei En, T. O’Coenenor, S. Parton, S. D. Robinson, M. I. Saat, B. N. Shurovi, K. Varatharasasingam, A. E. Ward, K. Behranwala, M. Bertelli, J. Cohen, F. Duff, O. Fafemi, R. Gupta, M. Manimaran, J. Mayhew, D. Peprah, M. H. Y. Wong, N. Farmer, C. Houghton, N. Kandhari, K. Khan, D. Ladha, J. Mayes, F. McLennan, P. Panahi, H. Seehra, R. Agrawal, I. Ahmed, S. Ali, F. Birkinshaw, M. Choudhry, S. Gokani, S. Harrogate, S. Jamal, F. Nawrozzadeh, A. Swaray, A. Szczap, J. Warusavitarne, M. Abdalla, N. Asemota, R. Cullum, M. Hartley, C. Maxwell-Armstrong, C. Mulvenna, J. Phillips, A. Yule, L. Ahmed, K. D. Clement, N. Craig, E. Elseedawy, D. Gorman, L. Kane, J. Livie, V. Livie, E. Moss, A. Naasan, F. Ravi, P. Shields, Y. Zhu, M. Archer, H. Cobley, R. Dennis, C. Downes, B. Guevel, E. Lamptey, H. Murray, A. Radhakrishnan, S. Saravanabavan, M. Sardar, C. Shaw, V. Tilliridou, R. Wright, W. Ye, N. Alturki, R. Helliwell, E. Jones, D. Kelly, S. Lambotharan, K. Scott, R. Sivakumar, L. Victor, H. Boraluwe-Rallage, P. Froggatt, S. Haynes, Y. M. A. Hung, A. Keyte, L. Matthews, E. Evans, P. Haray, I. John, A. Mathivanan, L. Morgan, O. Oji, C. Okorocha, A. Rutherford, H. Spiers, N. Stageman, A. Tsui, R. Whitham, A. Amoah-Arko, E. Cecil, A. Dietrich, H. Fitzpatrick, C. Guy, J. Hair, J. Hilton, L. Jawad, E. McAleer, Z. Taylor, J. Yap, M. Akhbari, D. Debnath, T. Dhir, M. Elbuzidi, M. Elsaddig, S. Glace, H. Khawaja, R. Koshy, K. Lal, L. Lobo, A. McDermott, J. Meredith, M. A. Qamar, A. Vaidya, F. Acquaah, L. Barfi, N. Carter, D. Gnanappiragasam, C. Ji, F. Kaminski, S. Lawday, K. Mackay, S. K. Sulaiman, R. Webb, P. Ananthavarathan, F. Dalal, E. Farrar, R. Hashemi, M. Hossain, J. Jiang, M. Kiandee, J. Lex, L. Mason, J. H. Matthews, E. McGeorge, S. Modhwadia, T. Pinkney, A. Radotra, L. Rickard, L. Rodman, A. Sales, K. L. Tan, A. Bachi, D. S. Bajwa, J. Battle, L. R. Brown, A. Butler, A. Calciu, E. Davies, I. Gardner, T. Girdlestone, O. Ikogho, G. Keelan, P. O’Loughlin, J. Tam, J. Elias, M. Ngaage, J. Thompson, S. Bristow, E. Brock, H. Davis, M. Pantelidou, A. Sathiyakeerthy, K. Singh, A. Chaudhry, G. Dickson, P. Glen, K. Gregoriou, H. Hamid, A. Mclean, P. Mehtaji, G. Neophytou, S. Potts, D. R. Belgaid, J. Burke, J. Durno, N. Ghailan, M. Hanson, V. Henshaw, U. R. Nazir, I. Omar, B. J. Riley, J. Roberts, G. Smart, K. Van Winsen, A. Bhatti, M. Chan, M. D’Auria, S. Green, C. Keshvala, H. Li, C. Maxwell-Armstrong, M. Michaelidou, L. Simmonds, C. Smith, A. Wimalathasan, J. Abbas, C. Cairns, Y. R. Chin, A. Connelly, S. Moug, A. Nair, D. Svolkinas, P. Coe, D. Subar, H. Wang, V. Zaver, J. Brayley, P. Cookson, L. Cunningham, A. Gaukroger, M. Ho, A. Hough, J. King, D. O’Hagan, A. Widdison, R. Brown, B. Brown, A. Chavan, S. Francis, L. Hare, J. Lund, N. Malone, B. Mavi, A. McIlwaine, S. Rangarajan, N. Abuhussein, H. S. Campbell, J. Daniels, I. Fitzgerald, S. Mansfield, A. Pendrill, D. Robertson, Y. W. Smart, T. Teng, J. Yates, A. Belgaumkar, A. Katira, J. Kossoff, S. Kukran, C. Laing, B. Mathew, T. Mohamed, S. Myers, R. Novell, B. L. Phillips, M. Thomas, T. Turlejski, S. Turner, M. Varcada, L. Warren, W. Wynell-Mayow, R. Church, L. Linley-Adams, G. Osborn, M. Saunders, R. Spencer, M. Srikanthan, S. Tailor, A. Tullett, M. Ali, S. Al-Masri, G. Carr, O. Ebhogiaye, S. Heng, S. Manivannan, J. Manley, L. E. McMillan, C. Peat, B. Phillips, S. Thomas, H. Whewell, G. Williams, A. Bienias, E. A. Cope, G. R. Courquin, L. Day, C. Garner, A. Gimson, C. Harris, K. Markham, T. Moore, T. Nadin, C. Phillips, S. M. Subratty, K. Brown, J. Dada, M. Durbacz, T. Filipescu, E. Harrison, E. D. Kennedy, E. Khoo, D. Kremel, I. Lyell, S. Pronin, R. Tummon, C. Ventre, L. Walls, E. Wootton, A. Akhtar, E. Davies, D. El-Sawy, M. Farooq, M. Gaddah, H. Griffiths, I. Katsaiti, N. Khadem, K. Leong, I. Williams, C. S. Chean, D. Chudek, H. Desai, N. Ellerby, A. Hammad, S. Malla, B. Murphy, O. Oshin, P. Popova, S. Rana, T. Ward, T. E. F. Abbott, O. Akpenyi, F. Edozie, R. El Matary, W. English, S. Jeyabaladevan, C. Morgan, V. Naidu, K. Nicholls, S. Peroos, J. Prowle, S. Sansome, H. D. Torrance, D. Townsend, J. Brecher, H. Fung, Z. Kazmi, P. Outlaw, K. Pursnani, N. Ramanujam, A. Razaq, M. Sattar, S. Sukumar, T. S. E. Tan, K. Chohan, S. Dhuna, T. Haq, S. Kirby, J. Lacy-Colson, P. Logan, Q. Malik, J. McCann, Z. Mughal, S. Sadiq, I. Sharif, C. Shingles, A. Simon, S. Burnage, S. S. N. Chan, A. R. J. Craig, J. Duffield, A. Dutta, M. Eastwood, F. Iqbal, F. Mahmood, W. Mahmood, C. Patel, A. Qadeer, A. Robinson, A. Rotundo, A. Schade, R. D. Slade, M. De Freitas, H. Kinnersley, E. McDowell, S. Moens-Lecumberri, J. Ramsden, T. Rockall, L. Wiffen, S. Wright, C. Bruce, V. Francois, K. Hamdan, C. Limb, A. J. Lunt, L. Manley, M. Marks, C. F. E. Phillips, C. J. F. Agnew, C. J. Barr, N. Benons, S. J. Hart, D. Kandage, R. Krysztopik, P. Mahalingam, J. Mock, S. Rajendran, M. T. Stoddart, B. Clements, H. Gillespie, S. Lee, R. McDougall, C. Murray, R. O’Loane, S. Periketi, S. Tan, R. Amoah, R. Bhudia, B. Dudley, A. Gilbert, B. Griffiths, H. Khan, N. McKigney, B. Roberts, R. Samuel, A. Seelarbokus, A. Stubbing-Moore, G. Thompson, P. Williams, N. Ahmed, R. Akhtar, E. Chandler, I. Chappelow, H. Gil, T. Gower, A. Kale, G. Lingam, L. Rutler, C. Sellahewa, A. Sheikh, H. Stringer, R. Taylor, H. Aglan, M. R. Ashraf, S. Choo, E. Das, J. Epstein, R. Gentry, D. Mills, Y. Poolovadoo, N. Ward, K. Bull, A. Cole, J. Hack, S. Khawari, C. Lake, T. Mandishona, R. Perry, S. Sleight, S. Sultan, T. Thornton, S. Williams, T. Arif, A. Castle, P. Chauhan, R. Chesner, T. Eilon, S. Kamarajah, C. Kambasha, L. Lock, T. Loka, F. Mohammad, S. Motahariasl, L. Roper, S. S. Sadhra, A. Sheikh, T. Toma, Q. Wadood, J. Yip, E. Ainger, S. Busti, L. Cunliffe, T. Flamini, S. Gaffing, C. Moorcroft, M. Peter, L. Simpson, E. Stokes, G. Stott, J. Wilson, J. York, A. Yousaf, A. Borakati, M. Brown, A. Goaman, B. Hodgson, A. Ijeomah, U. Iroegbu, G. Kaur, C. Lowe, S. Mahmood, Z. Sattar, P. Sen, A. Szuman, N. Abbas, M. Al-Ausi, N. Anto, R. Bhome, L. Eccles, J. Elliott, E. J. Hughes, A. Jones, A. S. Karunatilleke, J. S. Knight, C. C. F. Manson, I. Mekhail, L. Michaels, T. M. Noton, E. Okenyi, T. Reeves, I. H. Yasin, D. A. Banfield, R. Harris, D. Lim, C. Mason-Apps, T. Roe, J. Sandhu, N. Shafiq, E. Stickler, J. P. Tam, L. M. Williams, P. Ainsworth, Y. Boualbanat, C. Doull, E. Egan, L. Evans, K. Hassanin, G. Ninkovic-Hall, W. Odunlami, M. Shergill, M. Traish, D. Cummings, S. Kershaw, J. Ong, F. Reid, H. Toellner, A. Alwandi, M. Amer, D. George, K. Haynes, K. Hughes, L. Peakall, Y. Premakumar, N. Punjabi, A. Ramwell, H. Sawkins, J. Ashwood, A. Baker, C. Baron, I. Bhide, E. Blake, C. De Cates, R. Esmail, H. Hosamuddin, J. Kapp, N. Nguru, M. Raja, F. Thomson, H. Ahmed, G. Aishwarya, R. Al-Huneidi, S. Ali, R. Aziz, D. Burke, B. Clarke, A. Kausar, D. Maskill, L. Mecia, L. Myers, A. C. D. Smith, G. Walker, N. Wroe, C. Donohoe, D. Gibbons, P. Jordan, C. Keogh, A. Kiely, P. Lalor, M. McCrohan, C. Powell, M. Power Foley, J. Reynolds, E. Silke, O. Thorpe, J. Tseun Han Kong, C. White, Q. Ali, J. Dalrymple, Y. Ge, H. Khan, R. S. Luo, H. Paine, B. Paraskeva, L. Parker, K. Pillai, J. Salciccioli, S. Selvadurai, V. Sonagara, L. R. Springford, L. Tan, S. Appleton, N. Leadholm, Y. Zhang, D. Ahern, M. Cotter, S. Cremen, T. Durrigan, V. Flack, N. Hrvacic, H. Jones, B. Jong, K. Keane, P. R. O’Connell, J. O’sullivan, G. Pek, S. Shirazi, C. Barker, A. Brown, W. Carr, Y. Chen, C. Guillotte, J. Harte, A. Kokayi, K. Lau, S. McFarlane, S. Morrison, J. Broad, N. Kenefick, D. Makanji, V. Printz, R. Saito, O. Thomas, H. Breen, S. Kirk, C. H. Kong, A. O’Kane, M. Eddama, A. Engledow, S. K. Freeman, A. Frost, C. Goh, G. Lee, R. Poonawala, A. Suri, P. Taribagil, H. Brown, S. Christie, S. Dean, R. Gravell, E. Haywood, F. Holt, E. Pilsworth, R. Rabiu, H. W. Roscoe, S. Shergill, A. Sriram, A. Sureshkumar, L. C. Tan, A. Tanna, A. Vakharia, S. Bhullar, S. Brannick, E. Dunne, M. Frere, M. Kerin, K. Muthu Kumar, T. Pratumsuwan, R. Quek, M. Salman, N. Van Den Berg, C. Wong, J. Ahluwalia, R. Bagga, C. M. Borg, C. Calabria, A. Draper, M. Farwana, H. Joyce, A. Khan, M. Mazza, G. Pankin, M. S. Sait, N. Sandhu, N. Virani, J. Wong, K. Woodhams, N. Croghan, S. Ghag, G. Hogg, O. Ismail, N. John, K. Nadeem, M. Naqi, S. M. Noe, A. Sharma, S. Tan, F. Begum, R. Best, A. Collishaw, J. Glasbey, D. Golding, B. Gwilym, P. Harrison, T. Jackman, N. Lewis, Y. L. Luk, T. Porter, S. Potluri, M. Stechman, S. Tate, B. Walford, F. Auld, A. Bleakley, S. Johnston, C. Jones, J. Khaw, S. Milne, S. O’Neill, K. K. R. Singh, R. Smith, A. Swan, N. Thorley, S. Yalamarthi, Z. D. Yin, A. Ali, V. Balian, R. Bana, K. Clark, C. Livesey, G. McLachlan, M. Mohammad, N. Pranesh, C. Richards, F. Ross, M. Sajid, M. Brooke, J. Francombe, J. Gresly, S. Hutchinson, K. Kerrigan, E. Matthews, S. Nur, L. Parsons, A. Sandhu, M. Vyas, F. White, A. Zulkifli, L. Zuzarte, A. Al-Mousawi, J. Arya, S. Azam, A. Azri Yahaya, K. Gill, R. Hallan, C. Hathaway, I. Leptidis, L. McDonagh, S. Mitrasinovic, N. Mushtaq, N. Pang, G. B. Peiris, S. Rinkoff, L. Chan, E. Christopher, M. M. H. Farhan-Alanie, A. Gonzalez-Ciscar, C. J. Graham, H. Lim, K. A. McLean, H. M. Paterson, A. Rogers, C. Roy, D. Rutherford, F. Smith, G. Zubikarai, R. Al-Khudairi, M. Bamford, M. Chang, J. Cheng, C. Hedley, R. Joseph, B. Mitchell, S. Perera, L. Rothwell, A. Siddiqui, J. Smith, K. Taylor, O. Wroe Wright, H. K. Baryan, G. Boyd, H. Conchie, L. Cox, J. Davies, S. Gardner, N. Hill, K. Krishna, F. Lakin, S. Scotcher, J. Alberts, M. Asad, J. Barraclough, A. Campbell, D. Marshall, W. Wakeford, P. Cronbach, F. D’Souza, E. Gammeri, J. Houlton, M. Hall, A. Kethees, R. Patel, M. Perera, J. Prowle, M. Shaid, E. Webb, S. Beattie, M. Chadwick, O. El-Taji, S. Haddad, M. Mann, M. Patel, K. Popat, L. Rimmer, H. Riyat, H. Smith, C. Anandarajah, M. Cipparrone, K. Desai, C. Gao, E. T. Goh, M. Howlader, N. Jeffreys, A. Karmarkar, G. Mathew, H. Mukhtar, E. Ozcan, A. Renukanthan, N. Sarens, C. Sinha, A. Woolley, R. Bogle, O. Komolafe, F. Loo, D. Waugh, R. Zeng, A. Crewe, J. Mathias, A. Mills, A. Owen, A. Prior, I. Saunders, A. Baker, L. Crilly, J. McKeon, H. K. Ubhi, A. Adeogun, R. Carr, C. Davison, S. Devalia, A. Hayat, R. B. Karsan, C. Osborne, K. Scott, C. Weegenaar, M. Wijeyaratne, F. Babatunde, E. Barnor-Ahiaku, G. Beattie, P. Chitsabesan, O. Dixon, N. Hall, N. Ilenkovan, T. Mackrell, N. Nithianandasivam, J. Orr, F. Palazzo, M. Saad, L. Sandland-Taylor, J. Sherlock, T. Ashdown, S. Chandler, T. Garsaa, J. Lloyd, S. Y. Loh, S. Ng, C. Perkins, A. Powell-Chandler, F. Smith, R. Underhill, N. Goergen, A. McKinley, C. Neary, N. Rafiq, A. Badran, N. Fearnhead, M. Leadon, M. Yin Lin Ting, K. Conlon, D. Ganesan, D. O’Connor, M. J. Arthur, Z. Panayi, S. Rehman, H. Awni, R. Rao, A. Robinson, J. Baxter, P. Loughlin, A. Ahmed, H. Barrow, M. T. Liviu, G. Harinath, S. Raveendran, S. Sait, A. Ali, M. Latter, S. Udalov, M. Bergstrom, H. Tabry, E. West, S. Dindyal, C. Gao, H. Patel, M. Bath, K. Bevan, M. Bica, X. M. Chan, J. Lee, S. O’Donnell, M. Ravindran, E. Blessing, J. H. De Sousa Magalhaes, P. Jain, B. Campbell, R. Evans, S. Poo, C. Sanghera, N. Standfield, D. Karponis, A. Mehdi, R. Patel, J. O’Callaghan, A. Kumar, M. Saat, S. Davidson, A. Hylands, E. McKie, R. Hughes, J. Latter, E. Leung, P. Dos Santos Jorge, J. Saramunda, S. Vig, P. Serebriakoff, J. Wayman, S. K. Yen, M. Coleman, S. Leong, I. Sajid, T. Tolppa, A. Fawole, D. Kandola, A. Khan, F. Babatunde, A. Harlinska, K. Sasapu, A. D. Durham-Hall, G. Fowler, M. Glithero, T. Stringfellow, A. Tulloch, A. Bagchi, A. Grant, O. Onibere, M. Bews-Hair, N. Rajaraman, T. Agarwal, S. Rabinowicz, A. Radford, E. Pedlar, A. Raja, H. Rshaidat, P. Y. A. Aw, E. MacKle, E. Y. L. Yap, R. Charnley, L. A. M. Lim, M. Naylor, B. Stainer, N. Alseed, R. Amarasinghe, R. Rajagopal, P. Horgan, S. Sohrabi, A. Wilkinson, N. Liew, J. Smallwood, N. Walker, E. Mutengesa, T. Rankin, K. Waite, E. Robertson-Waters, S. Stanger, C. Thorn, A. Botha, A. Fowler, T. Suri, P. Vickers, S. Denley, W. Johnston, L. Jiao, A. Pain, K. Vutipongsatorn, A. Kale, R. S. Karri, K. Waite, C. Johnson, J. Smith, C. Walsh, N. Dewan, J. Prowle, K. Theodoropoulou, P. Jain, T. Nisar, A. Ali, L. Chung, J. Thomas, M. Abbas, Souradip Mookerjee, J. Pitt, E. Budd, T. Fung, M. Li, D. MacAfee, N. Havers, A. Kelkar, M. Hanson, R. Ingleton, N. Sukirthan, A. Chang, I. Eiben, M. Qamar, H. Javanmard, N. Watson, D. Bahadori, I. Bloom, G. Pike, J. Black, M. Metcalfe, A. Radhakrishnan, J. Seehra, K. Almeida, H. Amin, R. Holdsworth, J. Yeung, S. Gurjar, R. Jones, M. Patel, A. Alam, H. Ali, J. BannardSmith, R. Khaw, A. Rais, R. Ahluwalia, E. Briggs, H. Gil, J. Clements, R. Cowden, L. McCarthy, S. Chan, S. F. Hussain, R. Hryniv, H. Noble, J. Olivier, J. Prowle, S. Sait, E. Elseedawy, A. Hassane, I. Ibrahim, T. Melaugh, A. Ali, L. Ashraf, S. Green, K. Chapple, E. Heywood, N. Ngonyamo, I. Nyamali, A. Patil, T. Bamford, O. Fafemi, C. Grieco, K. Khan, A. Martin, H. Seehra, A. Burke-Smith, N. Johnson, G. Samarth, K. Sun, J. Warusavitarne, S. Green, C. Maxwell-Armstrong, J. Sivaraj, A. Campbell, M. Elseedawy, E. Elseedawy, O. Kouli, S. Bradbury, R. Dennis, H. Walji, J. Hale, P. Haray, P. Eiben, A. Light, T. Singhal, N. Carter, F. Ewbank, C. Perrott, I. Chappelow, R. Hashemi, A. Lee, J. Matthews, T. Pinkney, M. Byrne, H. Eltyeb, P. O’Loughlin, C. Donaldson, O. Oke, K. Bisset, P. Glen, S. Norman, L. Tan, M. Ahmed, C. Maxwell-Armstrong, S. Rangarajan, J. Sivaraj, C. Hancock, S. Moug, S. Smith, G. Nowell, B. Rigney, A. Widdinson, C. Boereboom, J. Lund, W. Simpson, J. Wright, I. Fitzgerald, S. Mansfield, E. Shakweh, K. Whitehurst, Z. Lee, B. Pinnell, G. Williams, R. Broll, T. Drake, E. Harrison, C. McCann, T. Abbott, S. Mahdi, F. Nawab, J. Prowle, B. Butcher, P. D. Loganathan, L. A. Paterson, K. Pursnani, J. Atley, K. Hamdan, E. Mills, B. Clements, G. Donaldson, L. Eaton, R. Aftab, M. Gough, B. Griffiths, C. Ng, G. Nolan, J. Archer, V. Do, S. Sharma, J. Epstein, P. Sodde, B. J. Storey, H. Ahmad, N. Akram, T. Sami, F. Sheldon, H. Croft, L. Han, K. Lasithiotakis, J. Acharya, O. Adeleye, G. Kaur, N. Dabab, P. Kangesu, J. Knight, K. Srikathrikamanathan, H. Wilson, E. Dell, L. Ellis, K. McDonald, D. Sobhanpanah, K. Foster, J. Mogg, S. Subramonia, P. Hill, A. Rahem, F. Reid, R. Bachar, N. Greenough, L. Hlukha, A. Ramwell, S. Carlton-Carew, M. Murray, A. Raja, D. Burke, M. El-Haddad, L. B. Mecia, N. Patel, R. Bhatt, W. J. Koay, L. Y. H. Low, J. Reynolds, S. Abbott, H. Devan Nair, J. J. Lee, R. O’Connell, W. Carr, S. Davies, S. Unsworth, J. Ashcroft, D. Lazenby, D. Subar, S. Choi, S. Rinkoff, N. Sarens, M. Varcada, N. Ellerby, A. Hammad, N. McCartan, U. Muhammad, M. Howlader, E. Norman, P. Polly, S. Brown, T. Clark, N. Thakral, P. Hann, R. Henderson, S. Kirk, S. Gupta, T. Richards, J. Ting, M. Byrne, C. Byrne, J. Cheema, S. Walsh, C. Borg, J. Hardie, Y. Sardar, B. Hughes, S. Saeed, F. Saeed, A. Sharma, E. Ang, B. Kansu, M. Stechman, R. Walford, C. Woodward, S. Adeyemi, R. Awad, L. Imam, I. Leptidis, E. D. Kennedy, H. Patterson, Z. M. Soh, L. Walls, J. D. Yau, B. Ali, D. Evans, J. Smith, E. James, V. E. Kantola, K. Krishna, H. Naeem, J. Prowle, O. Komolafe, E. Tilling, C. Osborne, J. Schuster Bruce, C. Weegenaar, P. Chitsabesan, A. Goaman, C. Goode, N. Nithianandavisam

**Affiliations:** Academic Department of Surgery, Heritage Building, University of Birmingham, Birmingham, UK

**Correction to: BMC Med Res Methodol**

**https://doi.org/10.1186/s12874-019-0857-y**

In the original publication of this article [1], the author should be only the collaboration group “STARSurg Collaborative”. In the “STARSurg Collaborative”, the author S. Mookerjee’s name should be shown as the full name Souradip Mookerjee.
