# Supplementary material for: Challenges of one-year longitudinal follow-up of a prospective, observational cohort study using an anonymised database: recommendations for trainee research collaboratives
Source: BMC Med Res Methodol. 2019 Dec 12;19:237. doi: 10.1186/s12874-019-0857-y (PMC6909648; doi:10.1186/s12874-019-0857-y)
Supplement: Supplementary file 1 — Additional file 1: Table S1. OAKS collaborator survey responses, centre activity and data completeness at one-year postoperatively. [file 12874_2019_857_MOESM1_ESM.docx]

**Table S1** OAKS collaborator survey responses, centre activity and data completeness at one-year postoperatively

|  | | | Respondent at a centre active in OAKS | | | | | |
| --- | --- | --- | --- | --- | --- | --- | --- | --- |
|  |  |  | **Active (n=**252**)** | | **Inactive (n=**33**)** | | **p-value** | |
| Stage of Training | Junior Doctor | 83 (87.4) | | 12 (12.6) | | 0.337 | |  |
|  | Later Year Student | 92 (86.0) | | 15 (14.0) | |  | |  |
|  | Early Year Student | 76 (92.7) | | 6 (7.3) | |  | |  |
| Previous OAKS collaborator | Yes | 117 (87.3) | | 17 (12.7) | | 0.596 | |  |
|  | No | 134 (89.3) | | 16 (10.7) | |  | |  |
| Prior experience with audit | Yes | 108 (86.4) | | 17 (13.6) | | 0.356 | |  |
|  | No | 143 (89.9) | | 16 (10.1) | |  | |  |
| Rating of experience identifying consultant | Positive (4-5) | 155 (93.4) | | 11 (6.6) | | 0.002 | |  |
|  | Not Positive (<4) | 96 (81.4) | | 22 (18.6) | |  | |  |
| Rating of experience registering audit * | Positive (4-5) | 118 (91.5) | | 11 (8.5) | | 0.138 | |  |
|  | Not Positive (<4) | 133 (85.8) | | 22 (14.2) | |  | |  |
| Rating of experience llinking Patient ID * | Positive (4-5) | 134 (95.7) | | 6 (4.3) | | <0.001 | |  |
|  | Not Positive (<4) | 117 (81.2) | | 27 (18.8) | |  | |  |
| Rating of experience collecting data * | Positive (4-5) | - | | - | | - | |  |
|  | Not Positive (<4) | - | | - | | - | |  |
